# Japonindolates A–C: new indole diterpenoids from the endophytic fungus *Aspergillus japonicus* 334 and their anti-inflammatory activities

**DOI:** 10.1039/d6ra08897j

**Published:** 2026-11-12

**Authors:** Li-Na Huo, Li-Juan Hu, Li-Na Wang, Gan Gu, Qing Yang, Zhen-Qing Sun

**Affiliations:** a Qingdao Hiser Hospital Affiliated of Qingdao University (Qingdao Traditional Chinese Medicine Hospital) Qingdao 266033 China szqhc1988@163.com 654774618@qq.com; b The Laboratory for Phytochemistry and Botanical Esticides, College of Agriculture, Jiangxi Agricultural University Nanchang 330045 China gugan@caas.cn

## Abstract

Chemical investigations on the endophytic fungus *Aspergillus japonicus* 334 yielded six structurally diverse indole diterpenoids (1–6) including three new compounds, namely japonindolates A–C (1–3). Their structures were elucidated by comprehensive spectroscopic analyses, including NMR, HRESIMS, electronic circular dichroism calculations, and quantum-chemical NMR calculations. The anti-inflammatory activities of compounds 1–6 were evaluated in lipopolysaccharide (LPS)-stimulated RAW264.7 macrophages and an LPS-induced inflammation model using *Tg(mpx:EGFP)* transgenic zebrafish larvae. In RAW264.7 macrophages, compounds 1–6 significantly reduced LPS-induced TNF-α secretion, compounds 2–4 and 6 decreased IL-1β secretion, and compounds 3–6 reduced NO production. In zebrafish larvae, compounds 2–4 and 6 significantly inhibited LPS-induced neutrophil recruitment, whereas compounds 1 and 5 showed no significant effects. Among the tested compounds, compound 4 showed the most pronounced activity across the tested inflammatory endpoints, followed by compound 6. These findings expand the chemical diversity of indole diterpenoids from endophytic fungi and highlight their potential as anti-inflammatory scaffolds for further investigation.

## Abstract 2

Six indole diterpenoids including three new compounds, which attenuated selected LPS-induced inflammatory responses in macrophage and zebrafish models, were reported.

## Graphical abstract

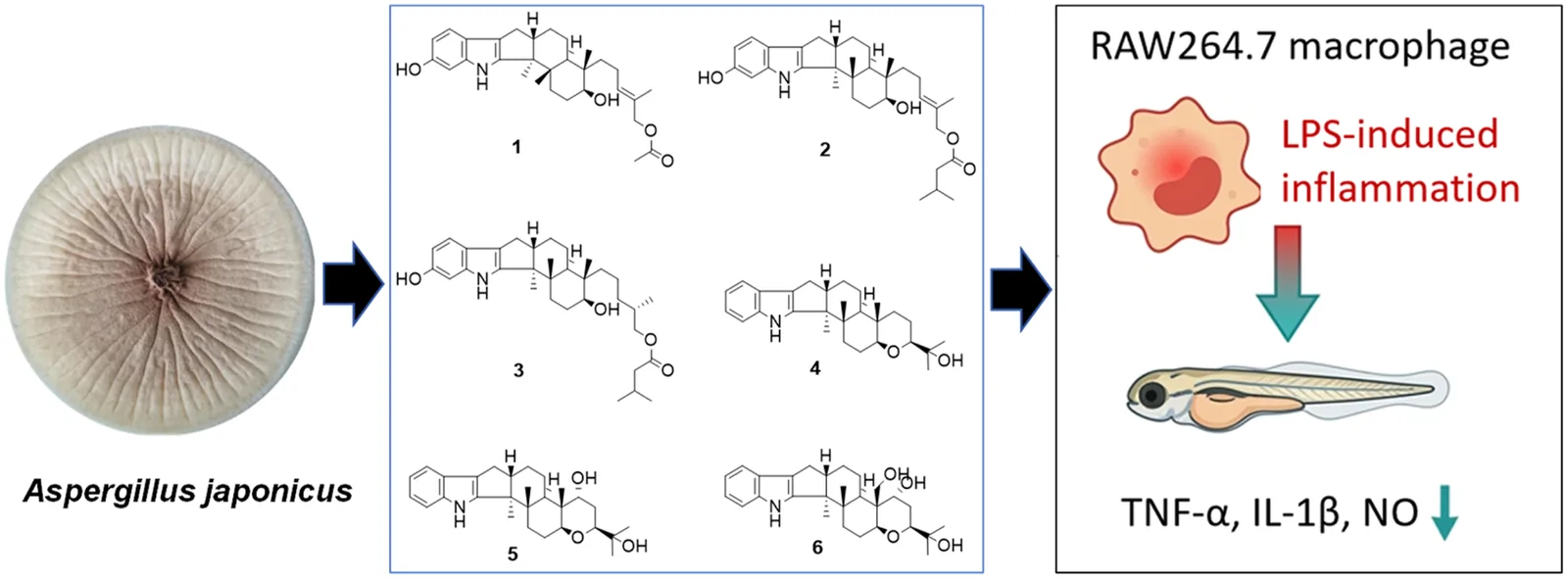

## 1 Introduction

Inflammation can protect the host from infection and tissue damage, whereas unresolved inflammation contributes to various chronic diseases.^1^ Promoting inflammatory resolution therefore represents an important therapeutic strategy.^2^ However, the adverse effects associated with prolonged use of NSAIDs (non-steroidal anti-inflammatory drugs) and glucocorticoid treatment highlight the need for alternative therapies.^3,4^ The complexity of inflammatory signaling further stimulates the exploration of compounds with distinct mechanisms of action.^5,6^

Natural products remain the primary source of therapeutic agents and provide structurally diverse scaffolds for the drug development.^7^ Their stereochemical complexity provides opportunities for interactions with diverse biological targets.^8^ Advances in metabolomics and molecular networking have facilitated their identification and prioritization.^9,10^ Importantly, natural products can modulate inflammatory cytokine production and NF-κB/MAPK signaling^11^ or inhibit inflammasome activation,^12,13^ supporting their continued investigation as anti-inflammatory lead compounds.

Endophytic fungi inhabit internal plant tissues without causing any apparent disease. They have proven to be a promising source of bioactive metabolites.^14^ Host-fungus interactions can influence their secondary metabolism,^15,16^ while fungal biosynthetic diversity further supports the discovery of novel secondary metabolites with various chemical scaffolds.^17^ Among these metabolites, indole diterpenoids display diverse structures and biological activities, making them attractive subjects for pharmacological investigation.^18,19^ In our previous studies, we identified structurally novel and biologically active metabolites from plant-associated endophytic fungi.^20–22^ However, the chemical diversity and anti-inflammatory potential of indole diterpenoids from endophytic *Aspergillus japonicus* remain insufficiently explored. In the present study, we investigated the secondary metabolites of *A. japonicus* 334, leading to the isolation of six indole diterpenoids (1–6), including three new compounds, namely japonindolates A–C (1–3) (Fig. 1). Their chemical structures were elucidated by spectroscopic and computational methods, and their anti-inflammatory properties were evaluated by using complementary macrophage and zebrafish inflammation models. By integrating chemical characterization with cell- and organism-level bioassays, this study provides new insights into the bioactive potential of indole diterpenoids from endophytic fungi.

**Fig. 1:**
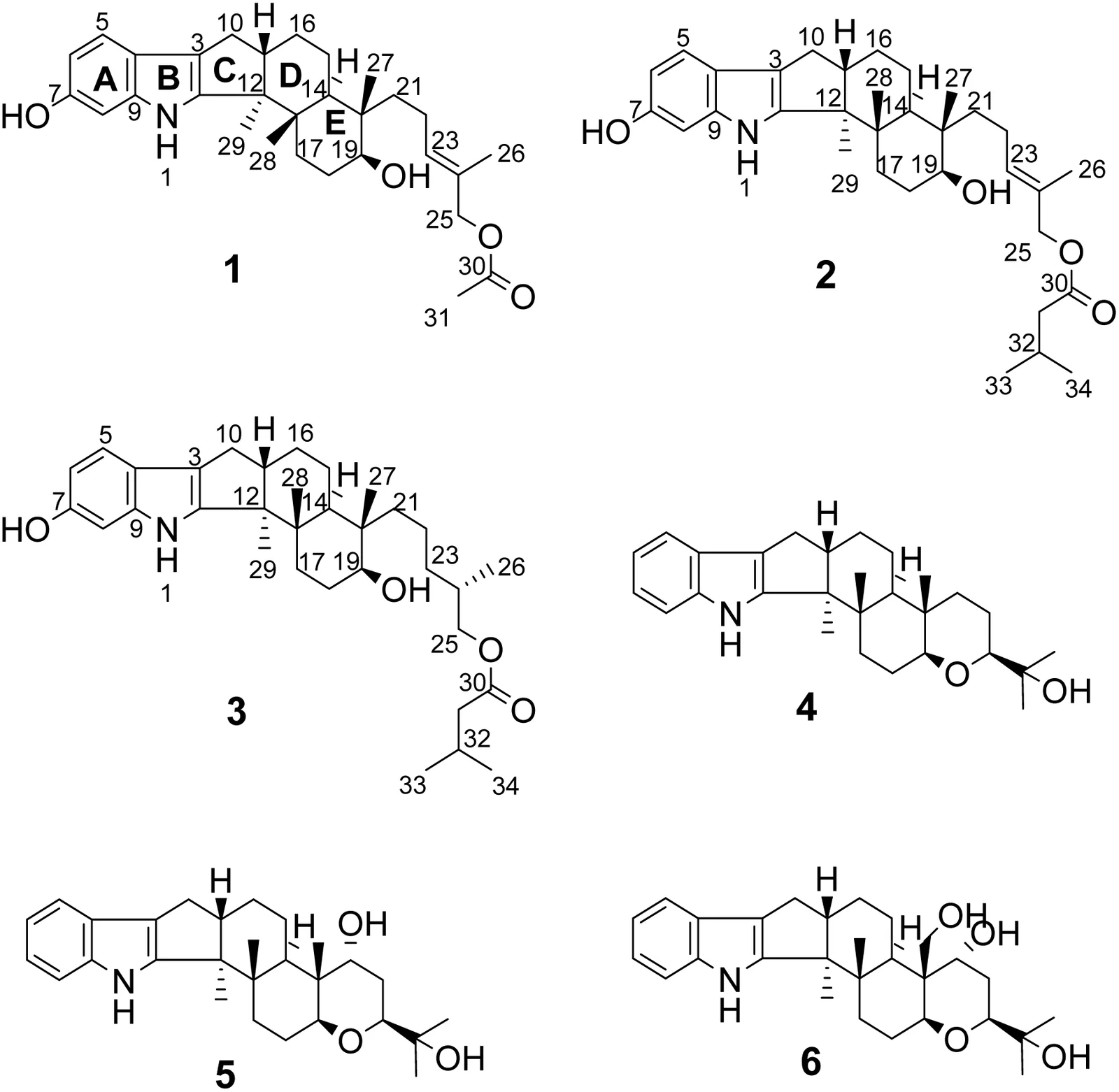
Structures of the isolated indole diterpenoids (1–6) from *Aspergillus japonicus* 334.

## 2 Results and discussion

### 2.1 Structure elucidation

Japonindolate A (1) was obtained as a pale yellow amorphous solid. Its molecular formula was determined to be C_30_H_41_NO_4_ on the basis of the HRESIMS data, which showed an [M + H]^+^ ion at *m*/*z* 480.3107 (calcd. for C_30_H_42_NO_4_^+^, 480.3108), corresponding to 11 degrees of unsaturation. The ^1^H NMR and HSQC spectra (Table 1) displayed three aromatic signals at *δ*_H_ 7.08 (d, *J* = 8.4 Hz, H-5), 6.51 (dd, *J* = 8.4, 2.0 Hz, H-6), and 6.74 (d, *J* = 2.0 Hz, H-8). Their coupling patterns were consistent with a 1,2,4-trisubstituted benzene ring. The spectra also exhibited an olefinic proton at *δ*_H_ 5.50 (t, *J* = 6.0 Hz, H-23), an oxygenated methylene signal at *δ*_H_ 4.46 (s, H_2_-25), and an oxygenated methine resonance at *δ*_H_ 3.53 (dd, *J* = 10.8, 4.8 Hz, H-19). Five methyl singlets were observed at *δ*_H_ 1.69 (s, H_3_-26), 0.82 (s, H_3_-27), 1.11 (s, H_3_-28), 0.99 (s, H_3_-29), and 2.05 (s, H_3_-31), together with a series of nonoxygenated aliphatic proton signals. The ^13^C NMR spectrum (Table 2) showed signals for a carbonyl carbon at *δ*_C_ 172.8, ten sp^2^ carbons at *δ*_C_ 152.8, 150.6, 143.2, 131.3, 130.9, 120.5, 119.0, 117.9, 109.4, and 98.7, and two oxygenated carbons at *δ*_C_ 74.0 and 71.4. The remaining resonances were assigned to seven methylenes, two methines, five methyls, and three aliphatic quaternary carbons. The carbonyl and sp^2^ carbon resonances accounted for six degrees of unsaturation, whereas the remaining five degrees of unsaturation required a pentacyclic system. These spectroscopic features suggested an indole diterpenoid framework.

**Table 1:** ^1^H NMR spectroscopic data for compounds 1–3 in CD_3_OD

Position	*δ*_H_ (*J* in Hz)		
	1	2	3
5	7.08 d (8.4)	7.09 d (8.4)	7.08 d (8.4)
6	6.51 dd (8.4, 2.0)	6.52 dd (8.4, 2.0)	6.50 dd (8.4, 2.0)
8	6.74 d (2.0)	6.75 d (2.0)	6.74 d (2.0)
10	2.56 dd (13.2, 6.6); 2.23 dd (13.2, 10.8)	2.55 dd (13.2, 6.6); 2.22 dd (13.2, 10.8)	2.55 dd (13.2, 6.6); 2.22 m
11	2.72 m	2.68 dddd (13.9, 10.8, 6.6, 3.2)	2.72 m
14	1.76 m	1.73 m	1.74 m
15	1.64 m; 1.46 m	1.59 m; 1.42 dt (13.2, 7.0)	1.59 m; 1.45 m
16	1.75 m; 1.63 m	1.72 m; 1.60 m	1.73 m; 1.61 m
17	1.81 m	1.79 m	1.79 m
18	1.85 m; 1.76 m	1.83 m; 1.75 m	1.80 m; 1.75 m
19	3.53 dd (10.8, 4.8)	3.52 dd (11.2, 4.4)	3.49 dd (10.8, 4.8)
21	1.64 m; 1.27 ddd (15.6, 12.0, 6.0)	1.63 m; 1.26 ddd (14.6, 11.9, 5.3)	1.55 m; 1.22 m
22	2.07 m; 1.99 tt (12.0, 6.0)	2.06 m; 1.97 dt (11.9, 6.2)	1.35 m; 1.24 m
23	5.50 t (6.0)	5.49 t (6.2)	1.39 m; 1.17 m
24			1.84 m
25	4.46 s	4.46 s	3.99 dd (10.8, 6.0); 3.89 dd (10.8, 6.6)
26	1.69 s	1.68 s	0.96 d (7.2)
27	0.82 s	0.81 s	0.82 s
28	1.11 s	1.08 s	1.11 s
29	0.99 s	0.96 s	0.98 s
31	2.05 s	2.20 m	2.21 m
32		2.07 m	2.08 m
33		0.95 d (6.8)	0.97 d (6.6)
34		0.95 d (6.8)	0.97 d (6.6)

**Table 2:** ^13^C NMR spectroscopic data for compounds 1–3 in CD_3_OD

Position	*δ*_C_		
	1^a^	2^b^	3^a^
2	150.6 C	150.5 C	150.6 C
3	117.9 C	117.8 C	117.8 C
4	120.5 C	120.5 C	120.5 C
5	119.0 CH	119.0 CH	119.0 CH
6	109.4 CH	109.4 CH	109.4 CH
7	152.8 C	152.7 C	152.8 C
8	98.7 CH	98.7 CH	98.7 CH
9	143.2 C	143.1 C	143.1 C
10	28.4 CH_2_	28.3 CH_2_	28.4 CH_2_
11	50.1 CH	50.0 CH	50.1 CH
12	54.4 C	54.3 C	54.4 C
13	40.4 C	40.4 C	40.3 C
14	41.3 CH	41.2 CH	41.3 CH
15	24.1 CH_2_	24.0 CH_2_	24.0 CH_2_
16	26.4 CH_2_	26.4 CH_2_	26.4 CH_2_
17	33.8 CH_2_	33.8 CH_2_	33.8 CH_2_
18	28.4 CH_2_	28.4 CH_2_	28.4 CH_2_
19	74.0 CH	74.0 CH	74.1 CH
20	42.5 C	42.4 C	42.4 C
21	37.9 CH_2_	37.9 CH_2_	38.9 CH_2_
22	22.3 CH_2_	22.3 CH_2_	20.8 CH_2_
23	131.3 CH	131.3 CH	35.4 CH_2_
24	130.9 C	130.9 C	33.8 CH
25	71.4 CH_2_	71.2 CH_2_	70.3 CH_2_
26	14.0 CH_3_	14.1 CH_3_	17.5 CH_3_
27	17.3 CH_3_	17.3 CH_3_	17.5 CH_3_
28	19.4 CH_3_	19.4 CH_3_	19.4 CH_3_
29	14.8 CH_3_	14.8 CH_3_	14.8 CH_3_
30	172.8 C	174.7 C	175.0 C
31	20.8 CH_3_	44.3 CH_2_	44.4 CH_2_
32		26.9 CH	27.0 CH
33		22.7 CH_3_	22.7 CH_3_
34		22.7 CH_3_	22.7 CH_3_

a Measured at 125 MHz ^13^C NMR.

b Measured at 150 MHz ^13^C NMR.

The planar structure of 1 was established from an analysis of the 2D NMR data (Fig. 2). The HMBC correlations from H-5 to C-4/C-7/C-8, from H-6 to C-4/C-7/C-8, and from H-8 to C-4/C-6/C-7/C-9, together with the H-5/H-6 cross-peak in the ^1^H–^1^H COSY spectrum and the *meta*-coupling observed between H-6 and H-8, supported the presence of the 1,2,4-trisubstituted benzene ring. Additional HMBC correlations from H-5 to C-3 and from H2-10 to C-2/C-3 indicated that C-2 and C-3 were conjugated with the aromatic ring through C-4. The molecular formula and the characteristic carbon chemical shifts further supported the assignment of a typical indole moiety.

**Fig. 2:**
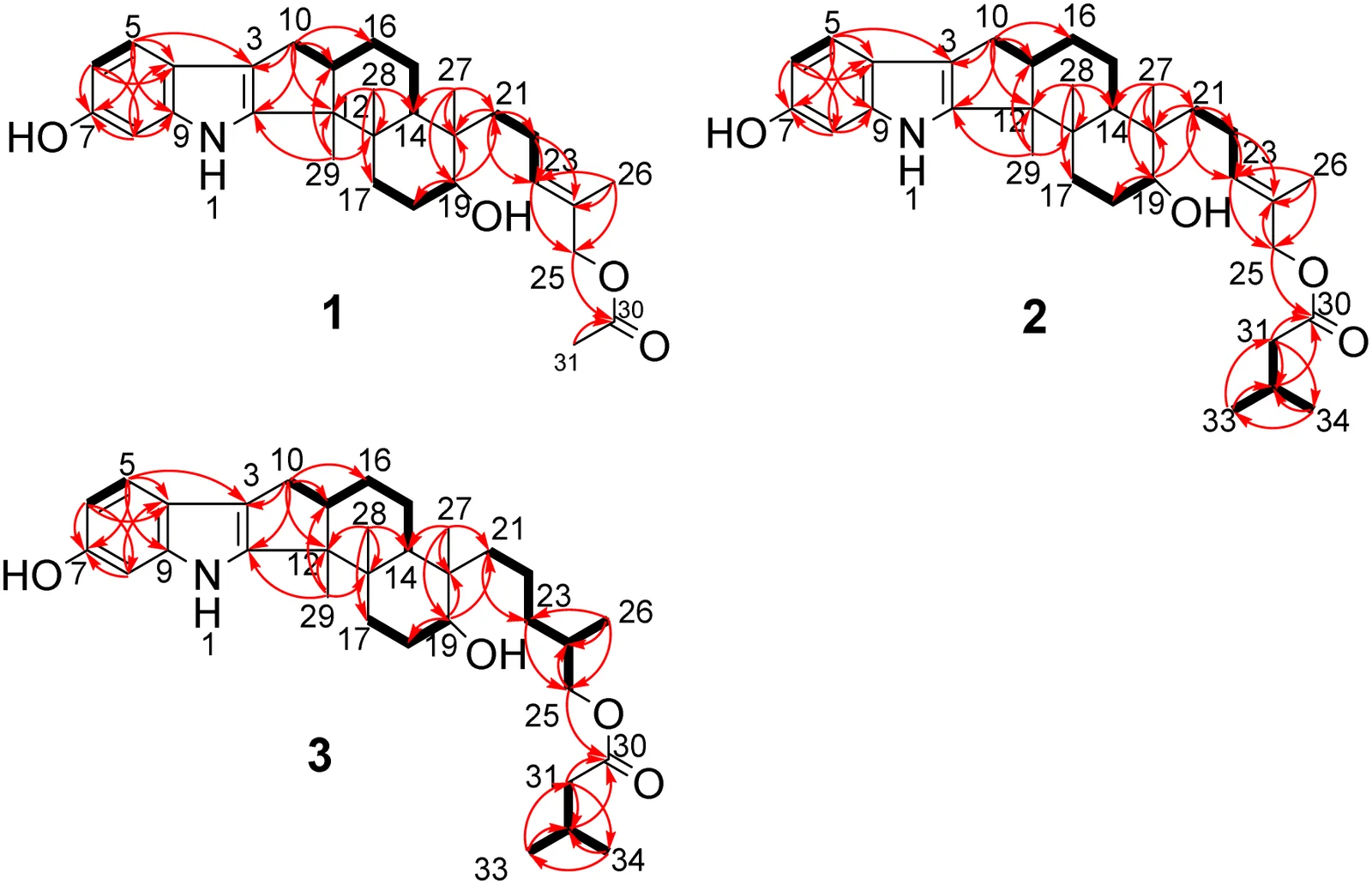
Key HMBC and ^1^H–^1^H COSY correlations of compounds 1–3.

A detailed comparison of the 2D NMR data showed that 1 closely resembled the paspaline-type indole diterpenoids (4).^23^ Compound 1 shared the same A–E ring system, but differed in the region corresponding to C-19 and C-23, where cleavage of the ether bridge gave rise to an acyclic side chain. The side-chain connectivity was established by the HMBC correlations from H_2_-21 to C-22/C-23, from H_2_-22 to C-21/C-23/C-24, from H_3_-26 to C-23/C-24/C-25, from H_2_-25 to C-23/C-26/C-30, and from H_3_-31 to C-30, together with the ^1^H–^1^H COSY correlations observed along the H_2_-21/H_2_-22/H-23 spin system. The attachment of this side chain to the diterpenoid core at C-20 was supported by HMBC correlations from H_3_-27 to C-14/C-19/C-20/C-21, from H-19 to C-21, and from H_2_-21 to C-19/C-20 (Fig. 2). These data established the planar structure of 1.

The relative configuration of 1 was assigned from the NOESY data (Fig. 3). NOESY correlations between H-11 and H_3_-28, and between H_3_-28 and H_3_-27, indicated that these protons and methyl groups were on the same face of the molecule, which was tentatively designated as the β-face. The NOESY correlations of H-11 with H-16eq (*δ*_H_ 1.75, m) and H-15ax (*δ*_H_ 1.46, m), together with those of H-14 with H-16ax (*δ*_H_ 1.63, m) and H-15eq (*δ*_H_ 1.64, m), supported the axial orientations of H-11 and H-14. Correlations between H_3_-29 and H-14, and between H-14 and H-19, indicated that H_3_-29, H-14, and H-19 were oriented toward the α-face. The NOESY correlations between H_2_-22 and H_3_-26 and between H-23 and H_2_-25 supported the E geometry of the C-23/C-24 double bond. Finally, comparison of the experimental ECD spectrum with the spectrum calculated for the proposed stereoisomer allowed the absolute configuration of 1 to be assigned as 11*S*,12*S*,13*S*,14*R*,19*S*,20*S* (Fig. 4A).

**Fig. 3:**
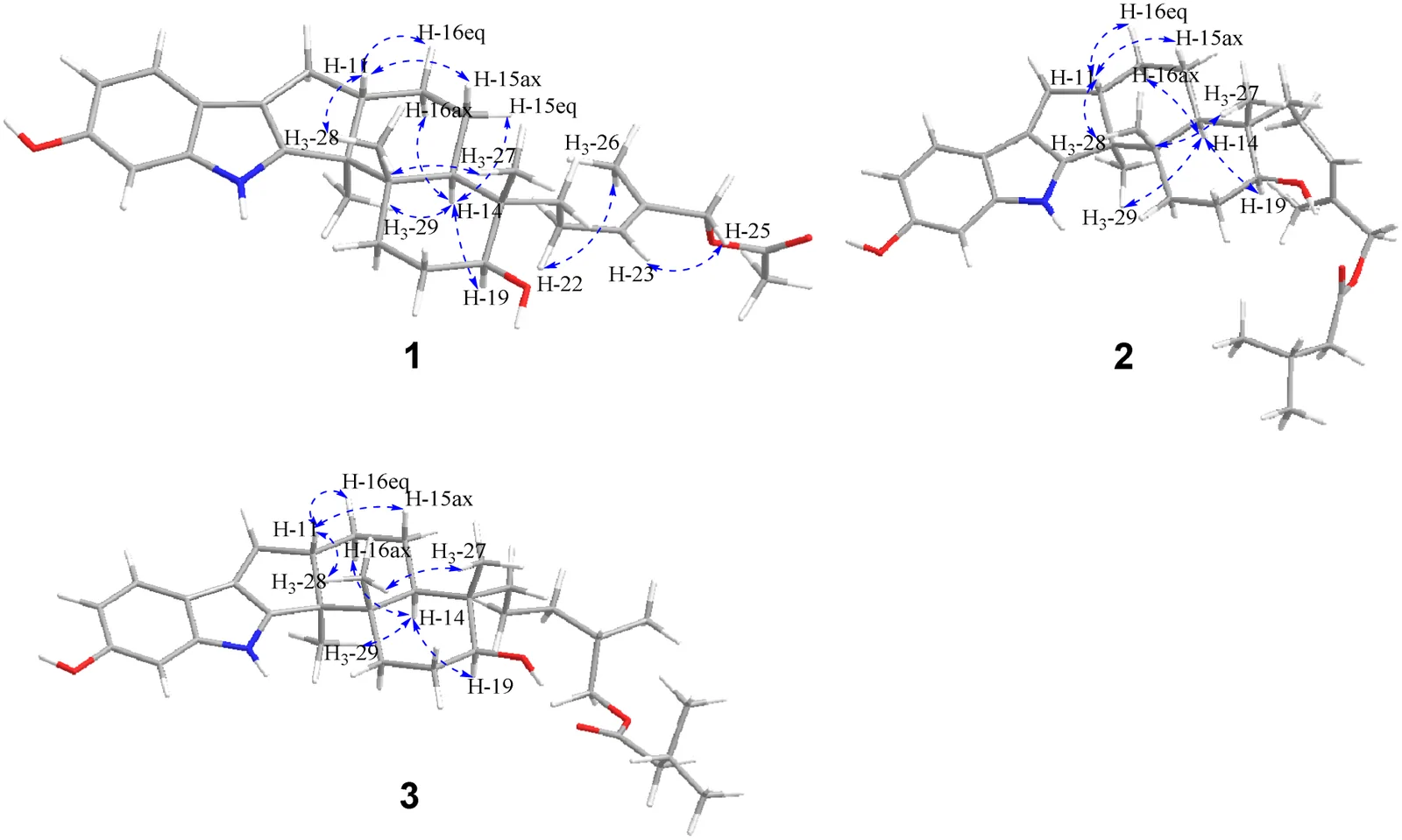
Key NOESY correlations of compounds 1–3.

**Fig. 4:**
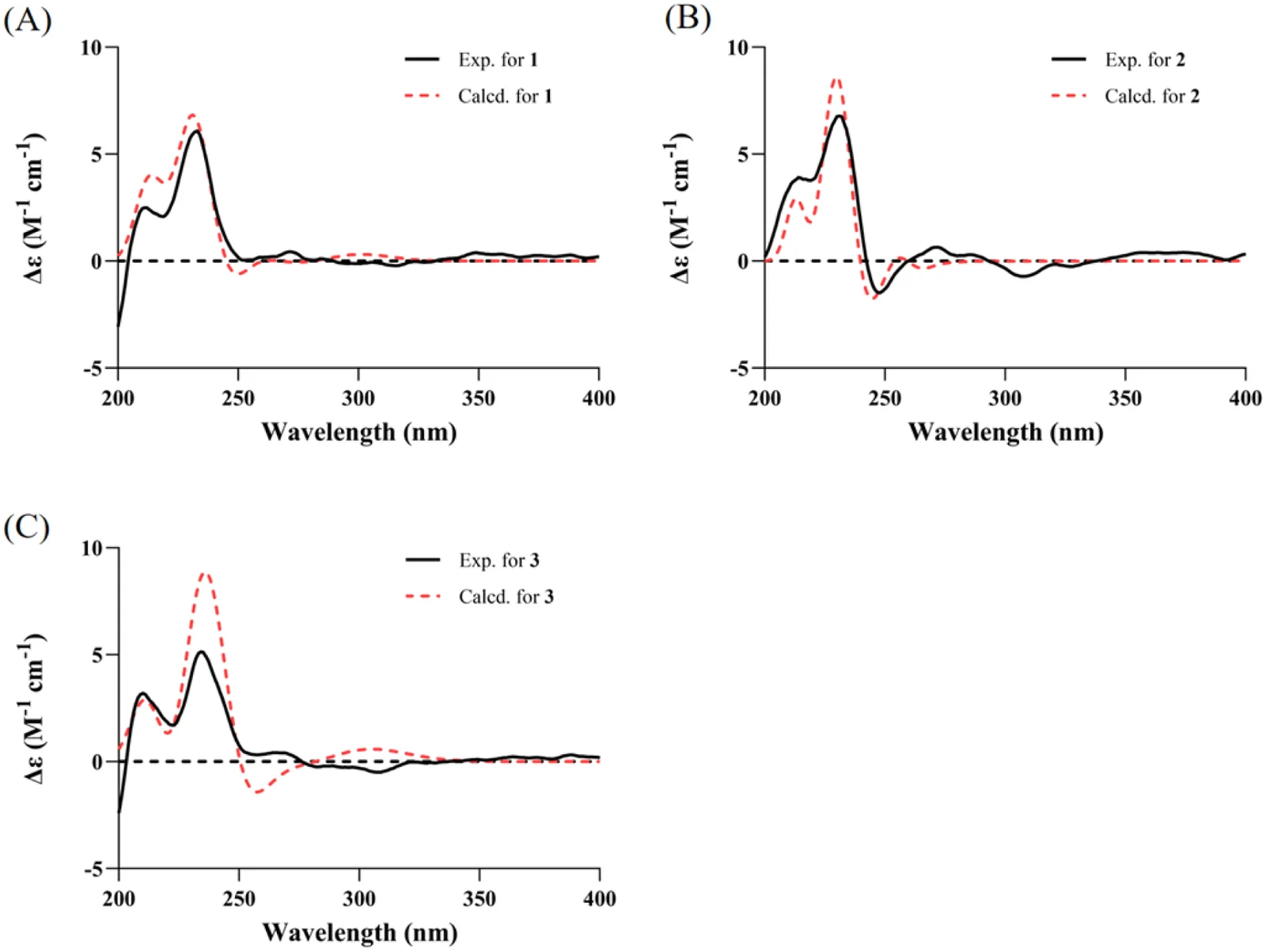
Experimental and calculated ECD spectra of compounds 1 (A), 2 (B), and 3 (C).

Japonindolate B (2) was isolated as a pale yellow amorphous solid. Its molecular formula was assigned as C_33_H_47_NO_4_ from the HRESIMS data, which showed an [M + H]^+^ ion at *m*/*z* 522.3578 (calcd for C_33_H_48_NO_4_^+^, 522.3578), corresponding to 11 degrees of unsaturation. The ^1^H and ^13^C NMR data of 2 (Tables 1 and 2) were closely comparable with those of 1, indicating that the two compounds possessed the same indole diterpenoid core. The principal difference was the acyl substituent at C-25: compound 2 contained a 3-methylbutanoyl group in place of the acetyl group present in 1. This assignment was supported by HMBC correlations from H_3_-33 to C-31/C-32/C-34, from H_3_-34 to C-31/C-32/C-33, from H-32 to C-30/C-31/C-33/C-34, and from H_2_-31 to C-30/C-32/C-33/C-34 (Fig. 2). Accordingly, the planar structure of 2 was established.

The relative configuration of 2 was deduced from its NOESY spectrum (Fig. 3). The observed correlations among H-11, H_3_-28, and H_3_-27, as well as those of H-11 with H-16eq and H-15ax, indicated that these nuclei were cofacial and were provisionally assigned to the β-face. The NOESY correlations of H_3_-29/H-14, H-14/H-19, and H-14/H-16ax supported the α-orientations of H_3_-29, H-14, and H-19. The ECD spectrum calculated by DFT for 11*S*,12*S*,13*S*,14*R*,19*S*,20*S*-2 at the B3LYP/6-31+G(d,p) level showed good agreement with the experimental ECD curve (Fig. 4B). Therefore, the absolute configuration of 2 was assigned as 11*S*,12*S*,13*S*,14*R*,19*S*,20*S*.

Japonindolate C (3) was obtained as a pale yellow amorphous solid. Its molecular formula was determined to be C_33_H_49_NO_4_ from the HRESIMS data, which gave an [M + H]^+^ ion at *m*/*z* 524.3734 (calcd for C_33_H_50_NO_4_^+^, 524.3730), corresponding to 10 degrees of unsaturation. The ^1^H and ^13^C NMR spectra of 3 (Tables 1 and 2) were similar to those of 2, except for the absence of the C-23/C-24 double bond. Instead, C-23 and C-24 were observed as saturated carbons in 3. This difference was supported by the HMBC correlations from H_3_-26 to C-23 (*δ*_C_ 35.4), C-24 (*δ*_C_ 33.8), and C-25 (*δ*_C_ 70.3), as well as by the ^1^H–^1^H COSY correlations along the H_2_-21/H_2_-22/H_2_-23/H-24/H_2_-25 spin system and between H_3_-26 and H-24 (Fig. 2).

The NOESY data indicated that the relative configurations at C-11, C-12, C-13, C-14, C-19, and C-20 were the same as those established for 2 (Fig. 3). However, the relative configuration of the additional stereogenic center at C-24 in the side chain could not be assigned unambiguously from the available NOESY data. To resolve this issue, GIAO-based ^13^C NMR chemical-shift calculations were performed using the mpw1pw91/6-311+G(2d,p) level with a methanol PCM solvent model, together with B3LYP/6-31+G(d,p) geometry optimization and the reported scaling parameters.^26^ The calculated chemical shifts for the 11S*,12S*,13S*,14R*,19S*,20S*,24S*-3 model showed better overall agreement with the experimental values than those calculated for the corresponding 24R* diastereomer (Fig. 5 and Table S1). Thus, the relative configuration at C-24 was assigned as S* relative to the established stereogenic centers. Finally, the ECD spectrum of compound 3 was calculated at the B3LYP/6-31+G(d) level. The calculated ECD spectrum showed good agreement with the experimental spectrum (Fig. 4C), supporting the assignment of the absolute configuration of compound 3 as 11*S*,12*S*,13*S*,14*R*,19*S*,20*S*,24*S*.

**Fig. 5:**
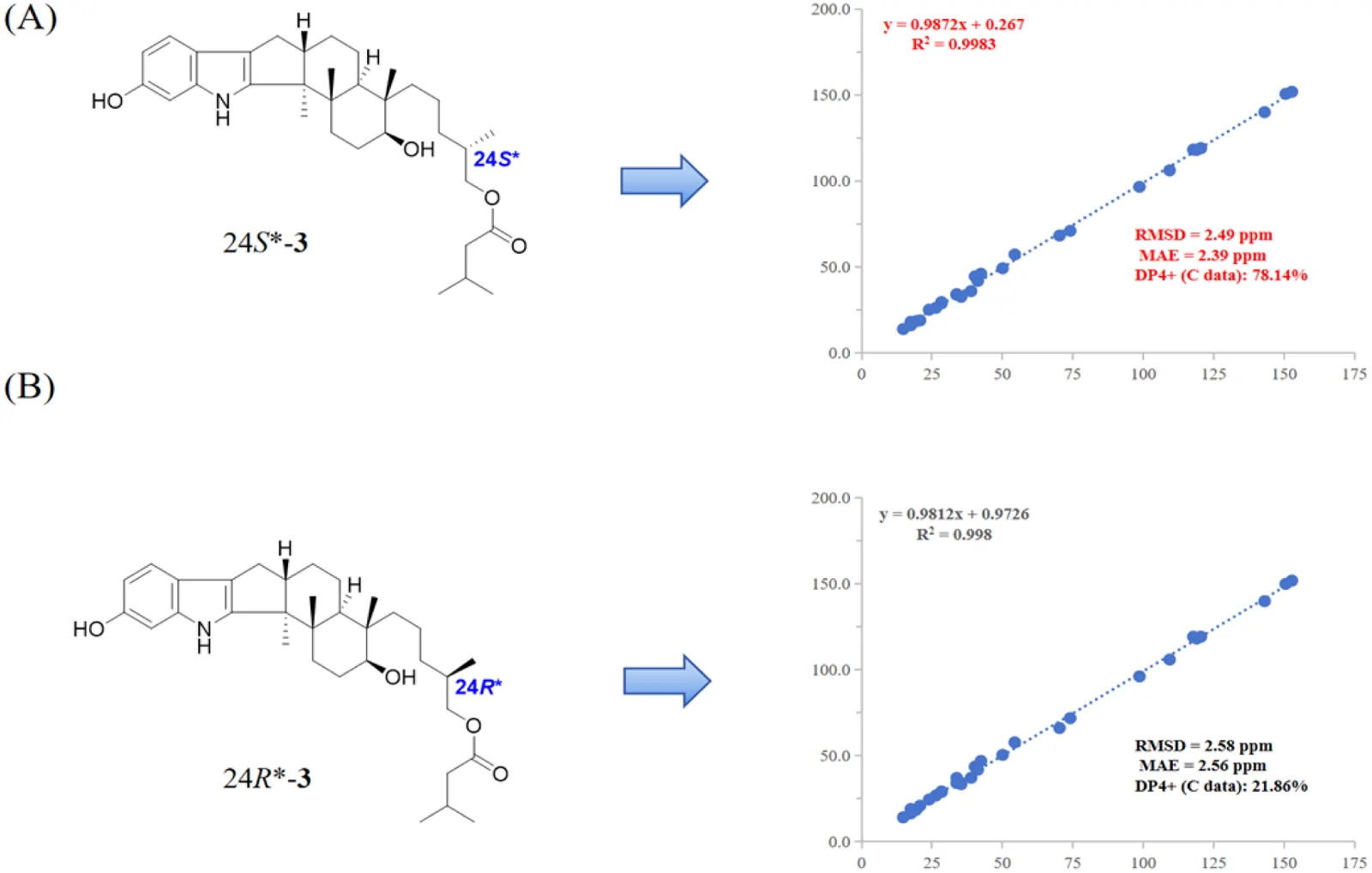
Comparison of the calculated ^13^C NMR chemical shifts for (A) 24S*-3 and (B) 24R*-3, with corresponding linear regression, RMSD, MAE, and DP4+ analyses.

Comparison of the NMR and HRESIMS data with published data led to the identification of compounds 4–6 as paspaline (4),^24^ terpendole E (5),^25^ and terpendole F (6),^25^ respectively.

In view of the structural diversity of the isolated indole diterpenoids and the reported biological activities of this class of metabolites, the anti-inflammatory properties of compounds 1–6 were subsequently evaluated *in vitro* and *in vivo*. An LPS-stimulated RAW264.7 macrophage model was first used to assess their effects on the production of the pro-inflammatory mediators TNF-α, IL-1β, and NO. The anti-inflammatory potential of the compounds was then further examined in an LPS-induced zebrafish inflammation model, together with an evaluation of their developmental toxicity.

### 2.2 *In vitro* anti-inflammatory activities of the isolated compounds in RAW264.7 cells

The effects of compounds 1–6 on RAW264.7 cell viability were first evaluated using the MTT assay to identify non-cytotoxic concentrations for subsequent anti-inflammatory experiments. Over the concentration range of 0.78–12.5 µg mL^−1^, none of the compounds caused a marked loss of cell viability, and the viability values generally remained close to or above 90% (Fig. 6A). At higher concentrations, particularly at 25 µg mL^−1^ and above, several compounds caused a significant reduction in cell viability. The most evident effects were observed for compounds 1, 4, and 6, whereas compound 3 showed a significant decrease mainly at 100 and 200 µg mL^−1^. Microscopic examination further showed that treatment with the compounds at 12.5 µg mL^−1^ did not cause apparent morphological abnormalities in RAW264.7 cells (Fig. 6B). Therefore, 12.5 µg mL^−1^ was selected as the highest common well-tolerated concentration for the subsequent *in vitro* anti-inflammatory assays. Microscopic examination further showed that treatment with the compounds at 12.5 µg mL^−1^ did not cause apparent morphological abnormalities in RAW264.7 cells (Fig. 6B). Accordingly, 12.5 µg mL^−1^ was selected as the highest common well-tolerated concentration for the subsequent *in vitro* anti-inflammatory assays. Concentrations associated with significant cytotoxicity were not used in these assays. Therefore, the observed decreases in inflammatory mediators are unlikely to be attributable to overt loss of cell viability.

**Fig. 6:**
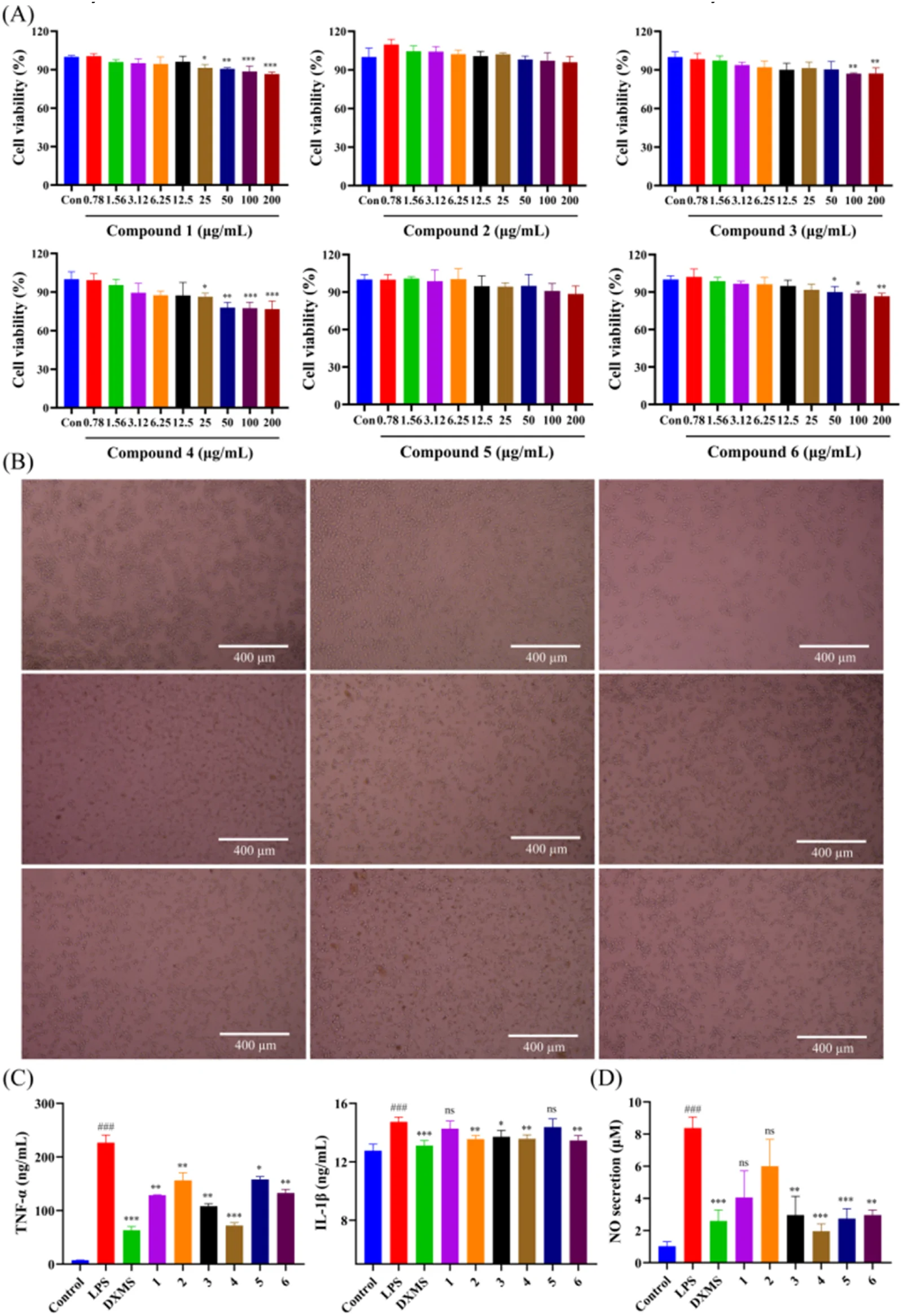
Effects of compounds 1–6 on inflammatory responses in lipopolysaccharide (LPS)-stimulated RAW264.7 macrophages. (A) Cytotoxic effects of the compounds 1–6 on RAW264.7 cells, as assessed by the MTT assay (*n* = 4); (B) representative morphological images of RAW264.7 macrophages treated with LPS in the presence of compounds 1–6 (scale bars = 400 µm); (C) levels of tumor necrosis factor-α (TNF-α) and interleukin-1β (IL-1β) in the culture supernatants of LPS-stimulated RAW264.7 macrophages (*n* = 4); (D) nitric oxide (NO) levels in culture supernatants (*n* = 4). ###*p* < 0.001 *versus* the control group; *p < 0.05, **p < 0.01, and ***p < 0.001 *versus* the LPS group; ns, not significant.

An LPS-stimulated RAW264.7 macrophage model was subsequently established to evaluate the anti-inflammatory effects of compounds 1–6. Compared with the untreated control group, LPS stimulation significantly increased the production of TNF-α, IL-1β, and NO (*p* < 0.001), confirming the successful induction of the inflammatory response (Fig. 6C and D). Dexamethasone (DXMS), which was used as the positive control at 10 µg mL^−1^, markedly suppressed the production of all three inflammatory mediators (*p* < 0.001).

Compounds 1–6 significantly reduced LPS-induced TNF-α production, although their inhibitory effects varied in magnitude. Compounds 1–3 and 6 showed significant inhibitory effects at *p* < 0.01, whereas compounds 4 and 5 inhibited TNF-α production at *p* < 0.001 and *p* < 0.05, respectively. Among the tested compounds, compound 4 exhibited the strongest inhibition of TNF-α production. Compounds 2–4 and 6 also significantly decreased IL-1β production, with *p* values of <0.01, <0.05, <0.01, and <0.01, respectively. In contrast, compounds 1 and 5 did not significantly affect IL-1β levels (Fig. 6C). Furthermore, compounds 3–6 significantly reduced NO production, with *p* values of <0.01, <0.001, <0.001, and <0.01, respectively, whereas compounds 1 and 2 showed no statistically significant effect on NO production. Taken together, compounds 3, 4, and 6 displayed the most consistent *in vitro* anti-inflammatory activities because they significantly suppressed the production of TNF-α, IL-1β, and NO in LPS-stimulated RAW264.7 macrophages. Compound 4 was particularly notable because it showed the strongest inhibitory effect against TNF-α and also markedly reduced IL-1β and NO production (Fig. 6D).

Like DXMS, compounds 3, 4, and 6 significantly suppressed all three measured mediators, whereas compounds 1, 2, and 5 affected only selected mediators. This similarity concerns the range of endpoints affected and does not establish equivalent inhibition or potency. DXMS and the isolated compounds were tested at 10 and 12.5 µg mL^−1^, respectively; concentration–response studies would be required to compare their relative potency.

### 2.3 *In vivo* anti-inflammatory activities of compounds 1–6 in zebrafish

The developmental toxicity of compounds 1–6 was first evaluated in zebrafish larvae. As shown in Table 3, no mortality was observed in any treatment group at concentrations of 0–25 µg mL^−1^, indicating that 25 µg mL^−1^ was a common concentration without observed mortality for compounds 1–6. At higher concentrations, compound-dependent increases in mortality were observed. Compound 1 caused mortality rates of 44.44%, 98.89%, and 100% at 50, 100, and 200 µg mL^−1^, respectively. Compounds 2 and 6 showed relatively lower toxicity at low concentrations, but their mortality rates increased to 45.56% and 98.89%, respectively, at 400 µg mL^−1^. Compounds 3, 4, and 5 also exhibited concentration-dependent toxicity, with mortality rates reaching 100%, 80.00%, and 100% at 400, 200, and 400 µg mL^−1^, respectively (Table 3). Representative morphological abnormalities, including pericardial edema, yolk retention, and swim bladder defects, were observed in larvae exposed to selected concentrations of the test compounds (Fig. 7A). The concentrations shown in Fig. 7A were selected to illustrate representative compound-associated morphological phenotypes at concentrations below or around those causing severe toxicity, rather than to represent equivalent doses across compounds. These concentrations were used for morphological toxicity assessment only and were not used in the subsequent anti-inflammatory assay. Based on the developmental toxicity results summarized in Table 3 25 µg mL^−1^ was selected as the highest common non-toxic concentration for evaluating the *in vivo* anti-inflammatory activities of compounds 1–6. No mortality or apparent morphological abnormalities were observed at this concentration for any of the six compounds, whereas higher concentrations caused compound-dependent toxicity. The concentration used in the zebrafish assay was higher than that used in the RAW264.7 cell assay because the two models showed distinct exposure conditions and toxicity profiles. Therefore, the selected concentrations were model-specific and should not be considered directly equivalent doses.

**Fig. 7:**
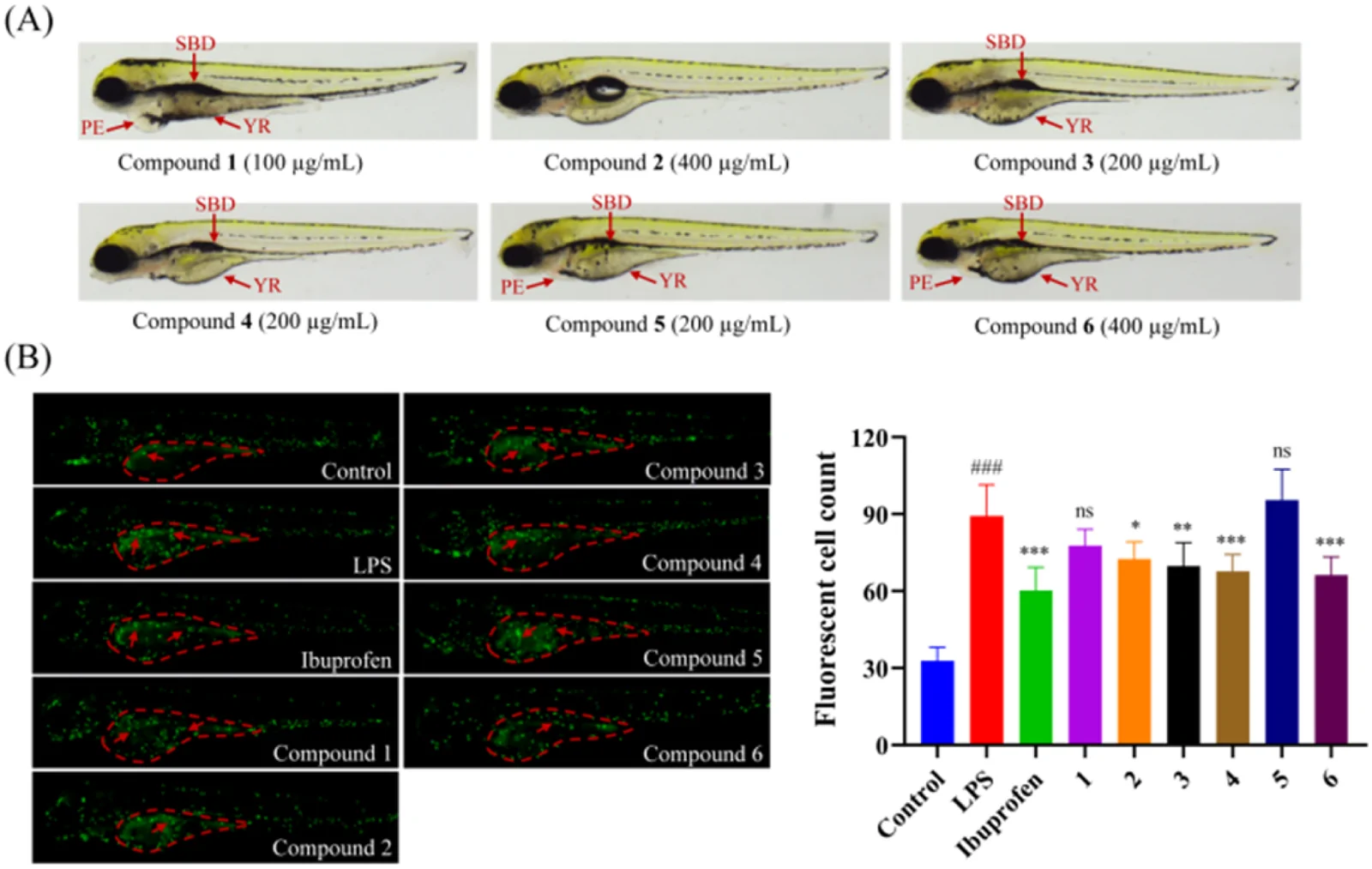
Effects of compounds 1–6 on LPS-induced inflammatory responses in zebrafish. (A) Representative images of developmental malformations in zebrafish larvae exposed to compounds 1–6 at the indicated concentrations. The concentrations were selected to illustrate representative compound-associated morphological phenotypes and were used for developmental toxicity assessment only; they were not used in the subsequent anti-inflammatory assay. SBD, swim bladder defect; YR, yolk retention; PE, pericardial edema; (B) effects of compounds 1–6 and ibuprofen on LPS-induced neutrophil recruitment in *Tg(mpx:EGFP)* zebrafish larvae (*n* = 7). ###*p* < 0.001 *versus* the control group; *p* < 0.05, *p < 0.01, and **p < 0.001 *versus* the LPS group; ns, not significant.

**Table 3:** Mortality of zebrafish larvae exposed to compounds 1–6. Data were obtained from three independent experiments, with 30 larvae per experiment (*N* = 90)

Compound (µg mL^−1^)	Zebrafish mortality rate (%)					
	1	2	3	4	5	6
0.78	0	0	0	0	0	0
12.5	0	0	0	0	0	0
25	0	0	0	0	0	0
50	44.44	0	6.67	18.89	0	0
100	98.89	0	34.44	52.22	21.11	36.67
200	100	0	63.33	80.00	92.22	68.89
400	100	45.56	100	100.00	100	98.89

An LPS-induced inflammation model was established in *Tg(mpx:EGFP)* transgenic zebrafish larvae by microinjecting 4 nL of LPS solution (0.5 mg mL^−1^) into the yolk sac. Compared with the control group, LPS stimulation markedly increased the accumulation of EGFP-positive neutrophils in the yolk sac, resulting in a significant increase in the fluorescent cell count (*p* < 0.001; Fig. 7B). Treatment with ibuprofen, which was used as the positive control, significantly reduced LPS-induced neutrophil recruitment (*p* < 0.001). At 25 µg mL^−1^, compounds 2, 3, 4, and 6 significantly inhibited LPS-induced neutrophil recruitment to the yolk sac, with *p* values of <0.05, <0.01, <0.001, and <0.001, respectively (Fig. 7B). Among these compounds, compounds 4 and 6 exhibited the most pronounced inhibitory effects. In contrast, compounds 1 and 5 did not significantly reduce the number of EGFP-positive neutrophils compared with the LPS-treated group.

Like ibuprofen, compounds 2, 3, 4, and 6 significantly inhibited neutrophil recruitment under the tested conditions. However, the significant responses relative to the LPS group do not establish comparable inhibition or potency between these compounds and ibuprofen. Ibuprofen was tested at 20 µM and the isolated compounds at 25 µg mL^−1^, and no concentration–response comparison was performed.

Compounds 1 and 5 showed selective effects in RAW264.7 cells: compound 1 reduced TNF-α, whereas compound 5 reduced TNF-α and NO; neither significantly altered IL-1β. Their different responses across the two models may reflect the distinct endpoints and model-specific test concentrations (12.5 µg mL^−1^ in cells and 25 µg mL^−1^ in zebrafish); differences in exposure or metabolism may also contribute, although these factors were not examined. The zebrafish result is limited to the single concentration tested.

Taken together, compounds 2, 3, 4, and 6 exhibited significant *in vivo* anti-inflammatory activity at the common nonlethal concentration of 25 µg mL^−1^, whereas compounds 1 and 5 showed no significant activity under the experimental conditions. Compounds 4 and 6 displayed the strongest inhibition of LPS-induced neutrophil recruitment, indicating that they were the most active compounds in the zebrafish inflammation model. Notably, the anti-inflammatory activity profiles observed in RAW264.7 macrophages were not completely consistent with those obtained in zebrafish larvae. For example, compounds 1 and 5 showed significant effects on selected inflammatory mediators in macrophages but did not significantly inhibit neutrophil recruitment in zebrafish. This discrepancy may reflect differences in compound exposure, metabolic stability, cellular targets, or the complexity of the inflammatory microenvironment. Therefore, the inhibitory effects observed in the macrophage assay may not necessarily translate directly into suppression of inflammatory responses in an intact organism.

It should be noted that the anti-inflammatory activities were evaluated at one selected, non-toxic concentration in each experimental model. Thus, these results provide a preliminary comparison of the activities of compounds 1–6, whereas detailed concentration–response relationships and potency parameters remain to be established.

## 3 Conclusions

In summary, chemical investigations on the endophytic fungus *A. japonicus* 334 led to the isolation and identification of six indole diterpenoids, including three previously undescribed compounds, japonindolates A–C (1–3), together with paspaline (4), terpendole E (5), and terpendole F (6). Their structures were established by integrated spectroscopic analyses, quantum-chemical NMR calculations, and electronic circular dichroism calculations.

The biological evaluation revealed that these compounds exerted distinct, rather than uniform, anti-inflammatory effects in both LPS-stimulated RAW264.7 macrophages and an LPS-induced zebrafish inflammation model. At 12.5 µg mL^−1^, all six compounds reduced TNF-α production, whereas compounds 2–4 and 6 additionally suppressed IL-1β secretion. Compounds 3–6 also inhibited NO production. Importantly, at the common non-lethal concentration of 25 µg mL^−1^, compounds 2–4 and 6 significantly attenuated LPS-induced neutrophil recruitment in zebrafish larvae, supporting the biological relevance of their anti-inflammatory effects in an intact organism. Among the tested compounds, paspaline (4) displayed the most consistent and pronounced activity across the evaluated endpoints, followed by terpendole F (6).

These results expand the chemical diversity of indole diterpenoids from endophytic fungi and provide preliminary evidence that structural variation within this scaffold can influence the profile and magnitude of anti-inflammatory activity. The differential responses observed among compounds 1–6 also suggest that the indole diterpenoid framework may serve as a useful starting point for further structure–activity relationship studies. Nevertheless, the molecular targets and intracellular pathways responsible for these effects remain to be clarified. Future studies should therefore focus on target identification, pathway-level validation, pharmacological optimization, and evaluation of the *in vivo* efficacy and safety of the most promising analogues.

## 4 Experimental

### 4.1 General experimental procedures

The general experimental procedures included multiple column chromatography, semi-preparative HPLC separation, and HPLC-DAD analysis, as well as the spectral measurements of the compounds. UV spectra were recorded using a UV2310II spectrophotometer (Techcomp, Ltd, Shanghai, China). Optical rotations (OR) were measured on a Rudolph Autopol IV automatic polarimeter (Rudolph Research Analytical, NJ, USA). Circular dichroism (CD) spectra were recorded on a JASCO J-1500 CD spectrometer (JASCO Corp., Tokyo, Japan). High-resolution electrospray ionization mass spectrometry (HRESIMS) spectra were acquired on a Micromass Q-TOF spectrometer (Waters, Milford, MA, USA). 1D and 2D NMR spectra (HSQC, HMBC, ^1^H–^1^H COSY, NOESY) were measured on a JNM-ECZ600R/S1 600 MHz NMR spectrometer (JEOL Ltd, Tokyo, Japan) and an Agilent DD2 500 MHz NMR spectrometer (Agilent Technologies, Santa Clara, CA, USA).

### 4.2 Fungal source

The fungal strain *A. japonicus* 334 was isolated in 2018 from healthy leaves of *Nicotiana tabacum* L. collected in Enshi City, Hubei Province, China. Primary isolation was performed on potato dextrose agar (PDA). For experimental use, the strain was grown on PDA plates (90 mm in diameter) at 28 °C for 5 days. Strain identification was performed by ITS rDNA sequence analysis. The ITS sequence was deposited in GenBank under accession number PV759848, and the isolate was identified as *A. japonicus*. A voucher culture designated as strain 334 was deposited at College of Agriculture, Jiangxi Agricultural University, Nanchang, China.

### 4.3 Fermentation, extraction and isolation

The activated strain *A. japonicus* 334 was first inoculated onto potato dextrose agar (PDA) plates under aseptic conditions and incubated at 28 °C for 5 days. Agar plugs measuring 1.0 × 1.0 cm were then excised from the actively growing cultures and transferred into 1 L Erlenmeyer flasks containing 300 mL of potato dextrose water (PDW) medium. The PDW medium was prepared using a commercial formulation (Haibo Biotechnology Co., Ltd, Qingdao, China; catalog no. HB0233-4) and sterilized by autoclaving at 121 °C for 20 min before inoculation.

The inoculated flasks were incubated under static conditions at room temperature for 30 days, with 12 h light and 12 h dark cycles per day. After fermentation, the culture broth was extracted ultrasonically with ethyl acetate for 30 min. The resulting organic extracts were concentrated to afford 146.5 g of crude extract. The crude extract was dissolved in MeOH and thoroughly mixed with silica gel (100–200 mesh) at a silica gel-to-crude extract ratio of 1 : 1 (w/w). The resulting material was subjected to vacuum silica gel column chromatography under reduced pressure, using a stepwise gradient of petroleum ether (PE) and EtOAc (100 : 10, 100 : 15, 100 : 20, 100 : 25, 100 : 35, 2 : 1, 1 : 1, 1 : 2, and 0 : 100, v/v). Nine fractions (Fr.1–Fr.9) were collected in order of increasing polarity. Fraction Fr.8, which was eluted with PE/EtOAc (1 : 2, v/v), was further separated by reversed-phase chromatography over a Lobar LiChroprep RP-18 column using a stepwise MeOH/H_2_O gradient from 50 : 50 to 100 : 0 (v/v; 300 mL per step). The eluates were concentrated under reduced pressure to give 23 subfractions (Fr.8.1–Fr.8.23).

Fraction Fr.8.22 was dissolved in MeOH, filtered, and purified by preparative HPLC on a Prep C_18_ column (5 µm, 10 × 150 mm) using an isocratic mixture of MeCN/H_2_O (65 : 35, v/v) containing 0.1% formic acid at a flow rate of 4 mL min^−1^. This purification yielded compounds 1 (2.3 mg, purity 96.3%), 2 (7.2 mg, purity 95.6%), and 3 (2.2 mg, purity 94.6%). Fraction Fr.8.20 was purified under the same HPLC conditions, except that MeCN/H_2_O (62 : 38, v/v) containing 0.1% formic acid was used as the isocratic mobile phase, to afford compounds 4 (3.6 mg, purity 94.3%), 5 (4.3 mg, purity 96.5%), and 6 (2.1 mg, purity 92.4%). The purity of compounds 1–6 was assessed by TLC and HPLC; the reported purity values were determined by HPLC analysis with UV detection at 210 nm.

Japonindolate A (1): yellow amorphous solid; [*α*]^25^_D_ +5.7 (*c* 0.10, MeOH); ECD (*c* = 5.22 × 10^−4^ M, MeOH) *λ* 208, 221, 234, 250, 275 nm; UV (MeOH) *λ*_max_: 231.8, 268.6, 304.3 nm; ^1^H NMR (CD_3_OD, 600 MHz) and ^13^C NMR (CD_3_OD, 125 MHz) provided in Tables 1 and 2; HRESIMS *m*/*z* 480.3107 [M + H]^+^ (calcd. for C_30_H_42_NO_4_^+^, 480.3108).

Japonindolate B (2): pale yellow amorphous solid; [*α*]^25^_D_ +7.1 (*c* 0.13, MeOH); ECD (*c* = 4.80 × 10^−4^ M, MeOH) *λ* 216, 222, 234, 248, 273 nm; UV (MeOH) *λ*_max_: 237.7, 269.7, 304.3 nm; ^1^H NMR (CD_3_OD, 600 MHz) and ^13^C NMR (CD_3_OD, 125 MHz) provided in Tables 1 and 2; HRESIMS *m*/*z* 522.3578 [M + H]^+^ (calcd. for C_33_H_48_NO_4_^+^, 522.3578).

Japonindolate C (3): yellow amorphous solid; [*α*]^25^_D_ +8.0 (*c* 0.10, MeOH); ECD (*c* = 4.78 × 10^−4^ M, MeOH) *λ* 209, 221, 236, 250, 271 nm; UV (MeOH) *λ*_max_: 230.6, 269.7, 305.5 nm; ^1^H NMR (CD_3_OD, 500 MHz) and ^13^C NMR (CD_3_OD, 150 MHz) provided in Tables 1 and 2; HRESIMS *m*/*z* 524.3730 [M + H]^+^ (calcd. for C_33_H_50_NO_4_^+^, 524.3734).

### 4.4 *In vitro* RAW264.7 cellular experiments

RAW264.7 cells were maintained in high-glucose DMEM supplemented with 10% fetal bovine serum (FBS) at 37 °C in a 5% CO_2_ atmosphere. For the cytotoxicity assay, the cells were seeded into 96-well plates at a density of 2 × 10^5^ cells mL^−1^ and allowed to adhere for 12 h. The cells were then treated with compounds 1–6 at concentrations of 0.78, 1.56, 3.12, 6.25, 12.5, 25, 50, 100, and 200 µg mL^−1^, with the corresponding vehicle-treated cells serving as the control. After 24 h of incubation, MTT assay was performed with DMSO as the solvent, and absorbance was measured at 490 nm to calculate cell viability. Based on the cytotoxicity results, 12.5 µg mL^−1^, which maintained acceptable cell viability for compounds 1–6, was selected as the working concentration for the subsequent anti-inflammatory assay.

For the anti-inflammatory assay, RAW264.7 cells were divided into the control, LPS model, dexamethasone (DXMS) positive-control, and compound-treatment groups. Except for the control group, the cells were stimulated with LPS at a final concentration of 1 µg mL^−1^. Cells in the positive-control group were treated with DXMS at 10 µg mL^−1^, whereas cells in the compound-treatment groups were treated with the corresponding isolated compound at 12.5 µg mL^−1^. After 24 h of incubation, the culture supernatants were collected for the determination of NO, TNF-α, and IL-1β levels. NO production was assessed by measuring nitrite levels using the Griess method, while TNF-α and IL-1β concentrations were determined using the corresponding commercial assay kits according to the manufacturers' instructions.

### 4.5 *In vivo* neutrophil recruitment assay with *Tg(mpx:EGFP)* transgenic zebrafish

Healthy *Tg(mpx:EGFP)* transgenic zebrafish larvae at 72 h postfertilization (hpf) were distributed into six-well plates at a density of 30 larvae per well. For the developmental toxicity assay, the larvae were exposed to vehicle or a series of concentrations of the test compounds ranging from 0 to 400 µg mL^−1^ at 28 °C for 24 h. Mortality and morphological abnormalities were recorded using a stereomicroscope. Based on the developmental toxicity results, 25 µg mL^−1^, which was the highest common concentration without apparent mortality or morphological abnormalities, was selected for the subsequent *in vivo* anti-inflammatory assay. This concentration was selected independently of the concentration used in the RAW264.7 assay because the two experimental models exhibited different exposure conditions and toxicity profiles.

For the neutrophil recruitment assay, the larvae were divided into four groups: the control, LPS model, ibuprofen positive-control, and compound-treatment groups. Ibuprofen was used at a concentration of 20 µM, whereas compounds 1–6 were each tested at 25 µg mL^−1^. Except for the control group, the larvae were microinjected with 4 nL of LPS solution (0.5 mg mL^−1^) into the yolk sac to induce inflammation. Following LPS microinjection, the larvae in the ibuprofen- and compound-treatment groups were exposed to the corresponding treatment for 6 h at 28 °C. The larvae were then examined under a stereoscopic fluorescence microscope, and EGFP-positive neutrophils recruited to the yolk sac were imaged and quantified. The number of fluorescent cells was used as an indicator of the *in vivo* anti-inflammatory activity of the tested compounds.

### 4.6 Ethical statement

All zebrafish experiments were conducted in accordance with relevant national and international guidelines for laboratory animal welfare and ethical research, including Directive 2010/63/EU of the European Parliament and of the Council on the protection of animals used for scientific purposes and the relevant provisions of Commission Delegated Directive (EU) 2024/1262 concerning animal housing, husbandry, and environmental conditions. The experiments exclusively involved zebrafish embryos at developmental stages prior to the onset of independent feeding, and no invasive procedures were performed. In accordance with Article 1(3) of Directive 2010/63/EU and the relevant provisions of Commission Implementing Decision (EU) 2020/569, zebrafish embryos at these pre-feeding stages are not considered protected experimental animals. Furthermore, phenotypic sex determination is not feasible at these early developmental stages. Therefore, formal approval from an institutional animal ethics committee was not required for this study.

### 4.7 Statistical analysis

All data were expressed as mean ± standard deviation (SD). Statistical significance was defined as *p* < 0.05. GraphPad Prism 8.3.0 was used for statistical analyses, and one-way ANOVA followed by post hoc tests was applied for multiple group comparisons.

## Author contributions

Li-Na Huo: writing – original draft, formal analysis. Li-Juan Hu: investigation, data curation, project administration. Li-Na Wang: methodology, investigation. Gan Gu: writing – review & editing, supervision, funding acquisition. Qing Yang: writing – review & editing, supervision. Zhen-Qing Sun: writing – review & editing, supervision.

## Conflicts of interest

There are no conflicts to declare.

## Supplementary Material

RA-OLF-D6RA08897J-s001

## Data Availability

The data supporting this article are available in the supplementary information (SI). Supplementary information: 1D and 2D NMR spectra, HRESIMS data, and UV spectra of compounds 1–3, together with the GIAO-based ^13^C NMR chemical-shift calculations and DP4+ analysisfor the two possible stereoisomers of compound 3 (Tables S1 and S2). See DOI: https://doi.org/10.1039/d6ra08897j.
