# Supplementary material for: Japonindolates A–C: new indole diterpenoids from the endophytic fungus Aspergillus japonicus 334 and their anti-inflammatory activities
Source: RSC Adv. 2026 Nov 12. Online ahead of print. doi: 10.1039/d6ra08897j (PMC13755754; doi:10.1039/d6ra08897j)
Supplement: Supplementary file 1 [file RA-OLF-D6RA08897J-s001.pdf]

Supplementary Information (SI) for RSC Advances.
This journal is © The Royal Society of Chemistry

Supporting Information

Japonindolates A–C: New indole diterpenoids from the endophytic fungus *Aspergillus japonicus* 334 and their anti-inflammatory activities

Li-Na Huo,[a] Li-Juan Hu,[a] Li-Na Wang,[a] Gan Gu,*[b] Qing Yang*[a] and Zhen-Qing Sun*[a]

[a] *Qingdao Hiser Hospital Affiliated of Qingdao University (Qingdao Traditional Chinese Medicine Hospital), Qingdao 266033, China*

[b] *The Laboratory for Phytochemistry and Botanical Pesticides, College of Agriculture, Jiangxi Agricultural University, Nanchang 330045, China*

Corresponding Author:

Gan Gu: The Laboratory for Phytochemistry and Botanical Pesticides, College of Agriculture, Jiangxi Agricultural University, Nanchang 330045, China; https://orcid.org/0000-0001-8163-327X; E-mail: gugan@caas.cn

Qing Yang: Qingdao Hiser Hospital Affiliated of Qingdao University (Qingdao Traditional Chinese Medicine Hospital), Qingdao 266033, China; https://orcid.org/0009-0002-4668-5976; E-mail: 654774618@qq.com

Zhen-Qing Sun: Qingdao Hiser Hospital Affiliated of Qingdao University (Qingdao Traditional Chinese Medicine Hospital), Qingdao 266033, China; https://orcid.org/0009-0007-8193-1200; E-mail: szqhc1988@163.com

Contents

Figure S1. ^{1}H NMR spectrum of **1** (CD_3OD, 600 MHz) ... 3

Figure S2. ^{13}C NMR spectrum of **1** (CD_3OD, 125 MHz) ... 3

Figure S3. HSQC spectrum of **1** ... 4

Figure S4. ^{1}H-^{1}H COSY spectrum of **1** ... 4

Figure S5. HMBC spectrum of **1** ... 5

Figure S6. NOESY spectrum of **1** ... 5

Figure S7. UV spectrum of **1** (100% MeOH/H_2O, extracted from HPLC-DAD data) ... 6

Figure S8. HRESIMS spectrum of **1** ... 6

Figure S9. ^{1}H NMR spectrum of **2** (CD_3OD, 500 MHz) ... 7

Figure S10. ^{13}C NMR spectrum of **2** (CD_3OD, 150 MHz) ... 7

Figure S11. HSQC spectrum of **2** ... 8

Figure S12. ^{1}H-^{1}H COSY spectrum of **2** ... 8

Figure S13. HMBC spectrum of **2** ... 9

Figure S14. NOESY spectrum of **2** ... 9

Figure S15. UV spectrum of **2** (100% MeOH/H_2O, extracted from HPLC-DAD data) ... 10

Figure S16. HRESIMS spectrum of **2** ... 10

Figure S17. ^{1}H NMR spectrum of **3** (CD_3OD, 600 MHz) ... 11

Figure S18. ^{13}C NMR spectrum of **3** (CD_3OD, 125 MHz) ... 11

Figure S19. HSQC spectrum of **3** ... 12

Figure S20. ^{1}H-^{1}H COSY spectrum of **3** ... 12

Figure S21. HMBC spectrum of **3** ... 13

Figure S22. UV spectrum of **3** (100% MeOH/H_2O, extracted from HPLC-DAD data) ... 13

Figure S23. HRESIMS spectrum of **3** ... 14

Table S1. GIAO ^{13}C NMR calculation of two potential isomers of japonindolate C (**3**) ... 15

Table S2. ^{13}C NMR-based DP4+ analysis of the two candidate stereoisomers of japonindolate C (**3**): Isomer 1, 24*S*-3; Isomer 2, 24*R*-3 ... 16

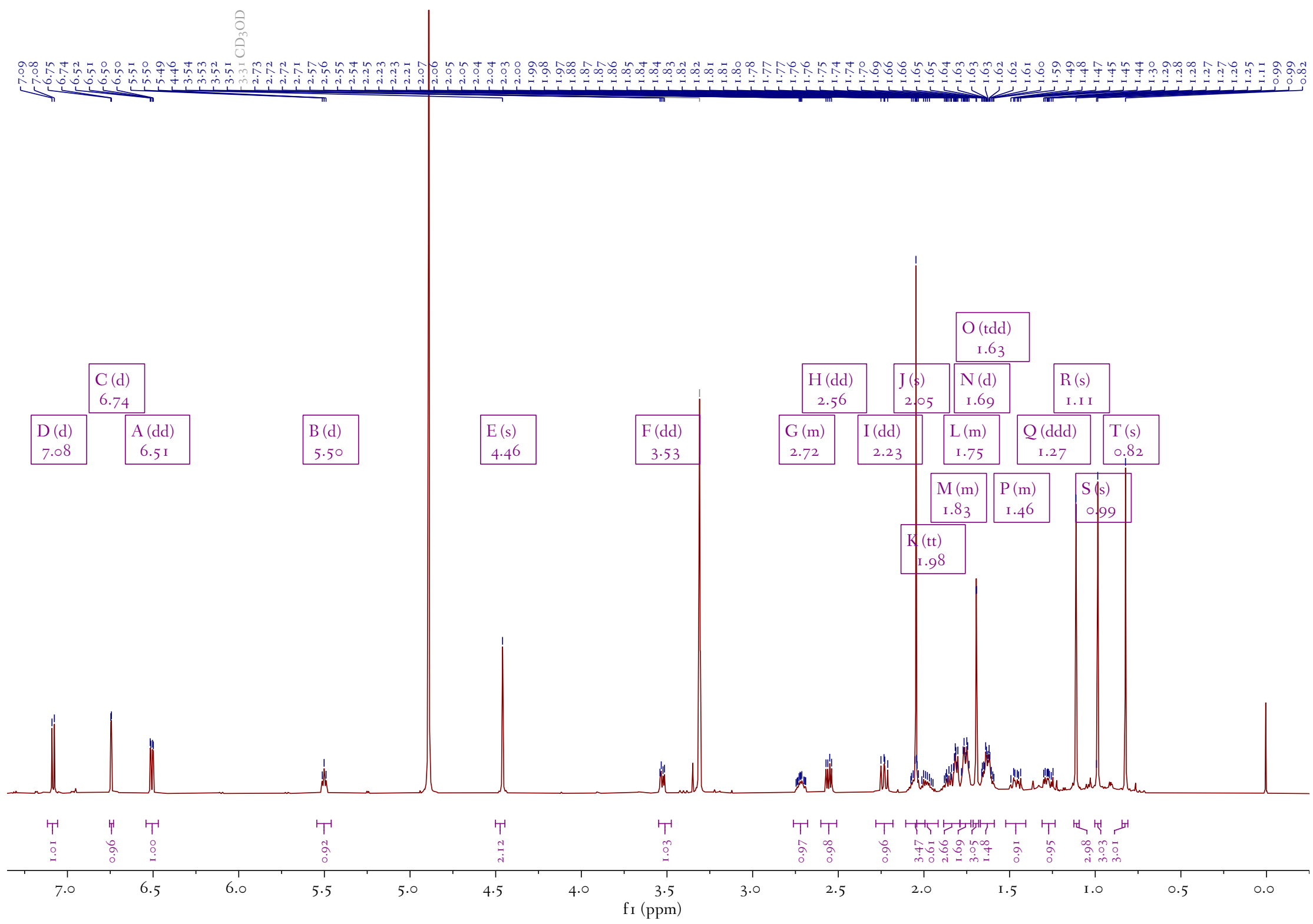

Figure S1. ^{1}H NMR spectrum of **1** (CD_3OD, 600 MHz)

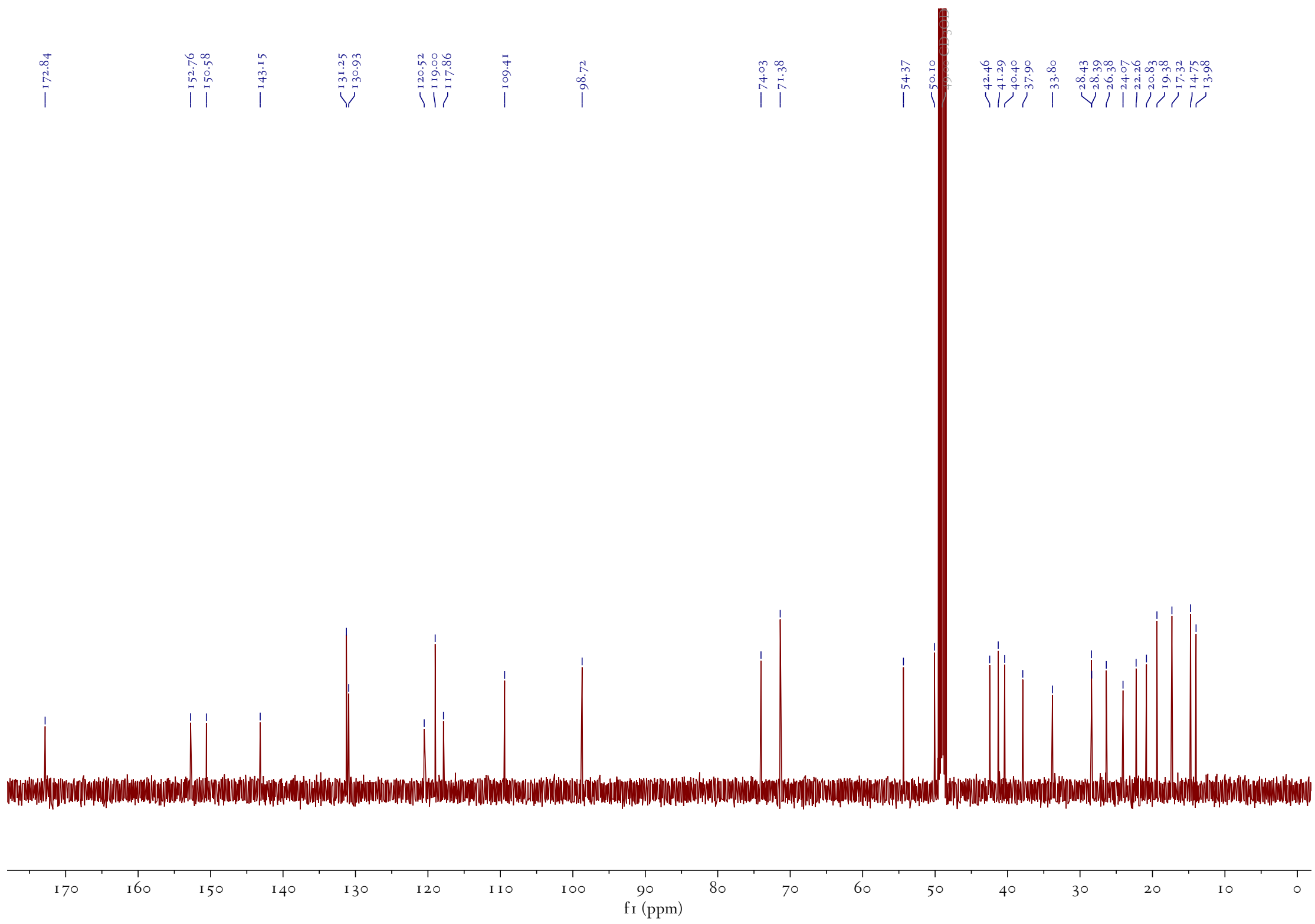

Figure S2. ^{13}C NMR spectrum of **1** (CD_3OD, 125 MHz)

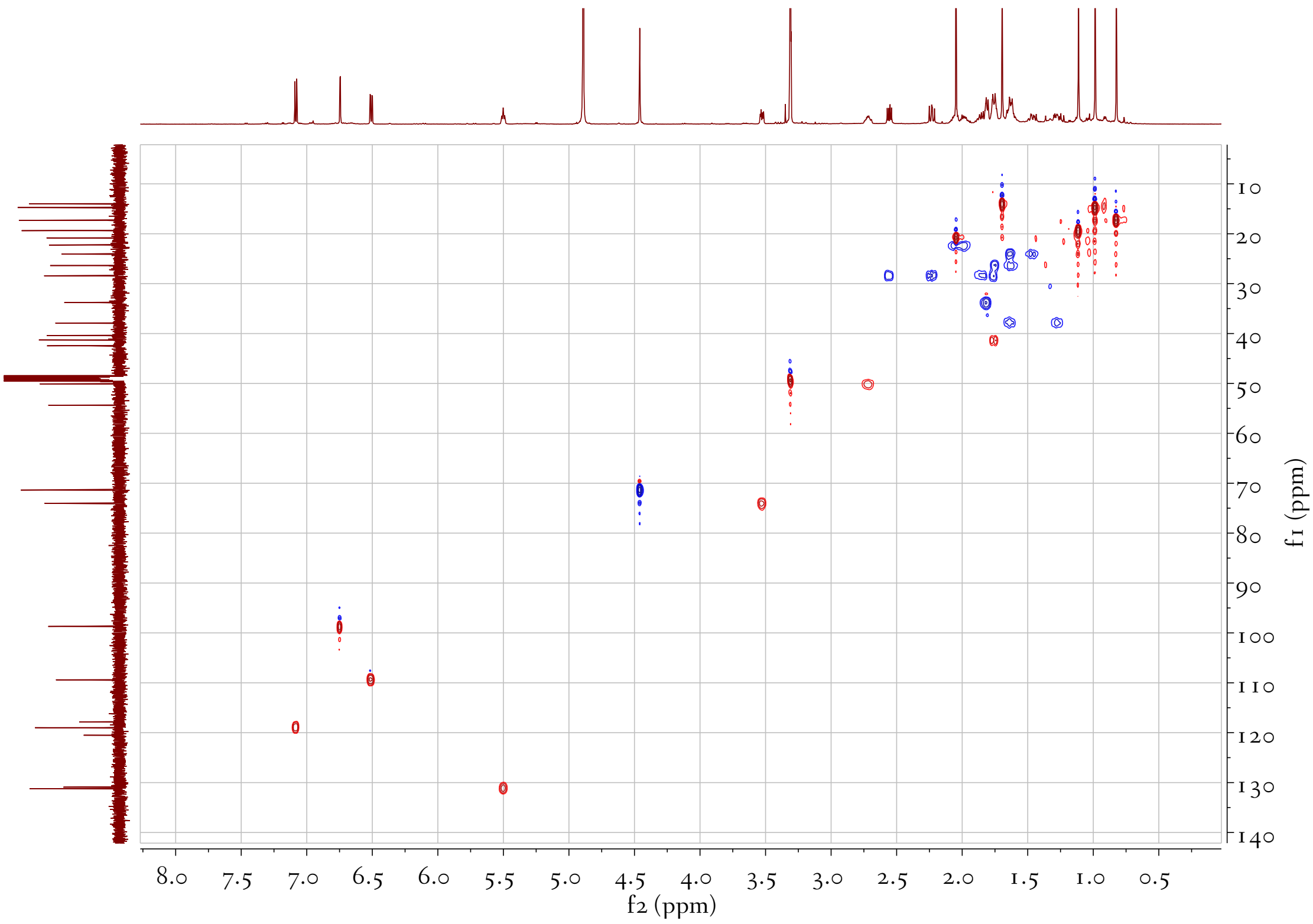

Figure S3. HSQC spectrum of **1**

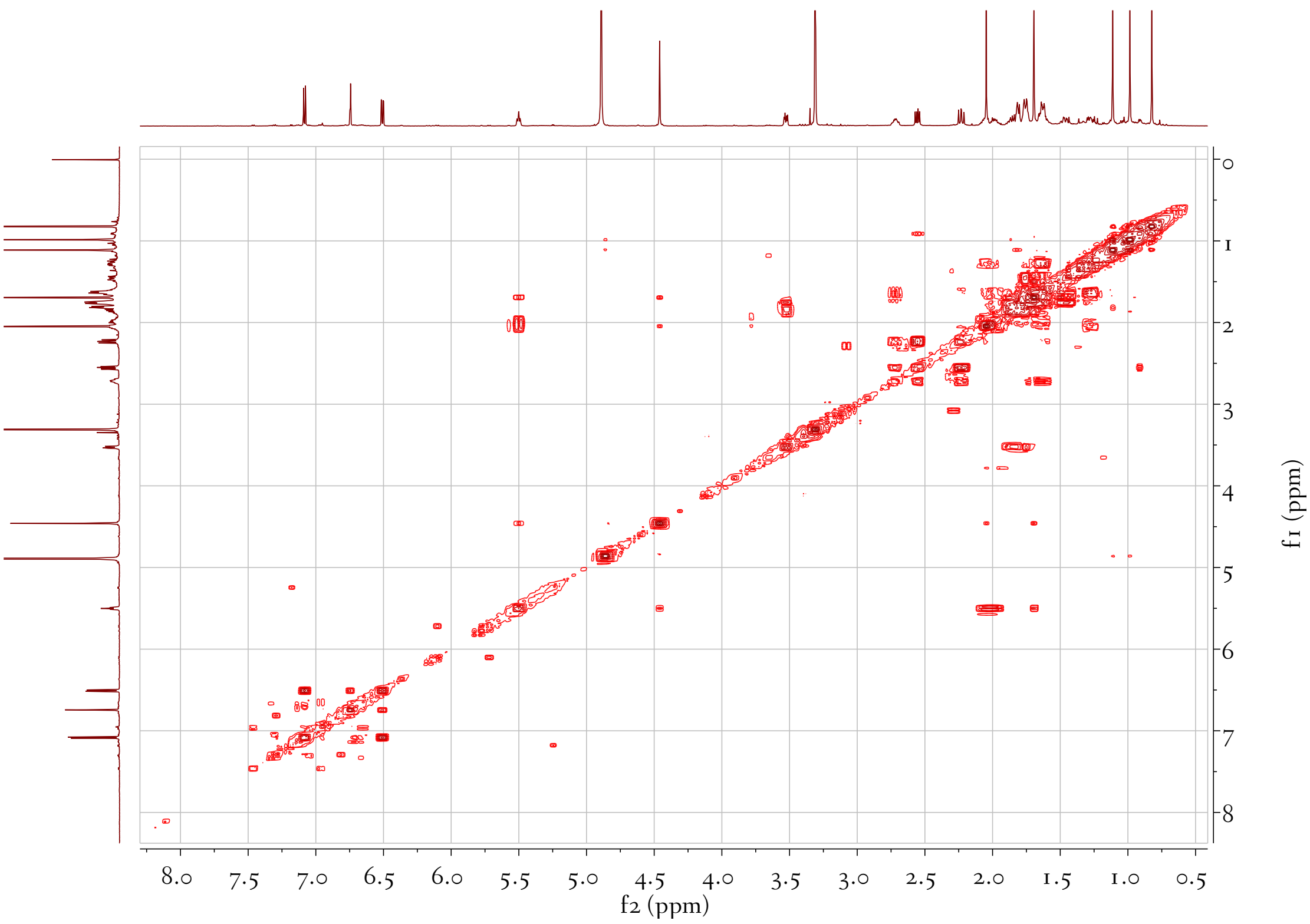

Figure S4. ^{1}H-^{1}H COSY spectrum of **1**

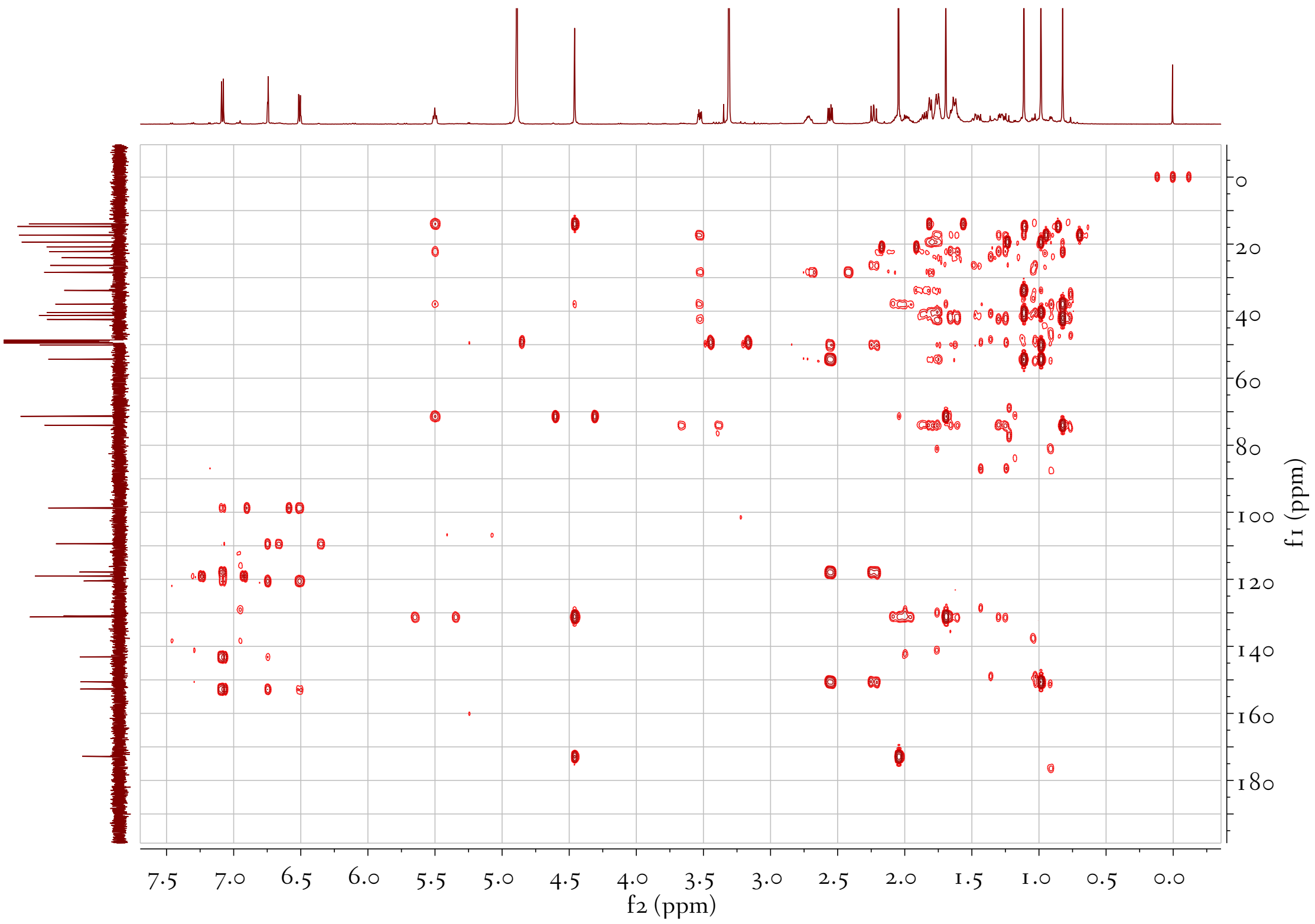

Figure S5. HMBC spectrum of **1**

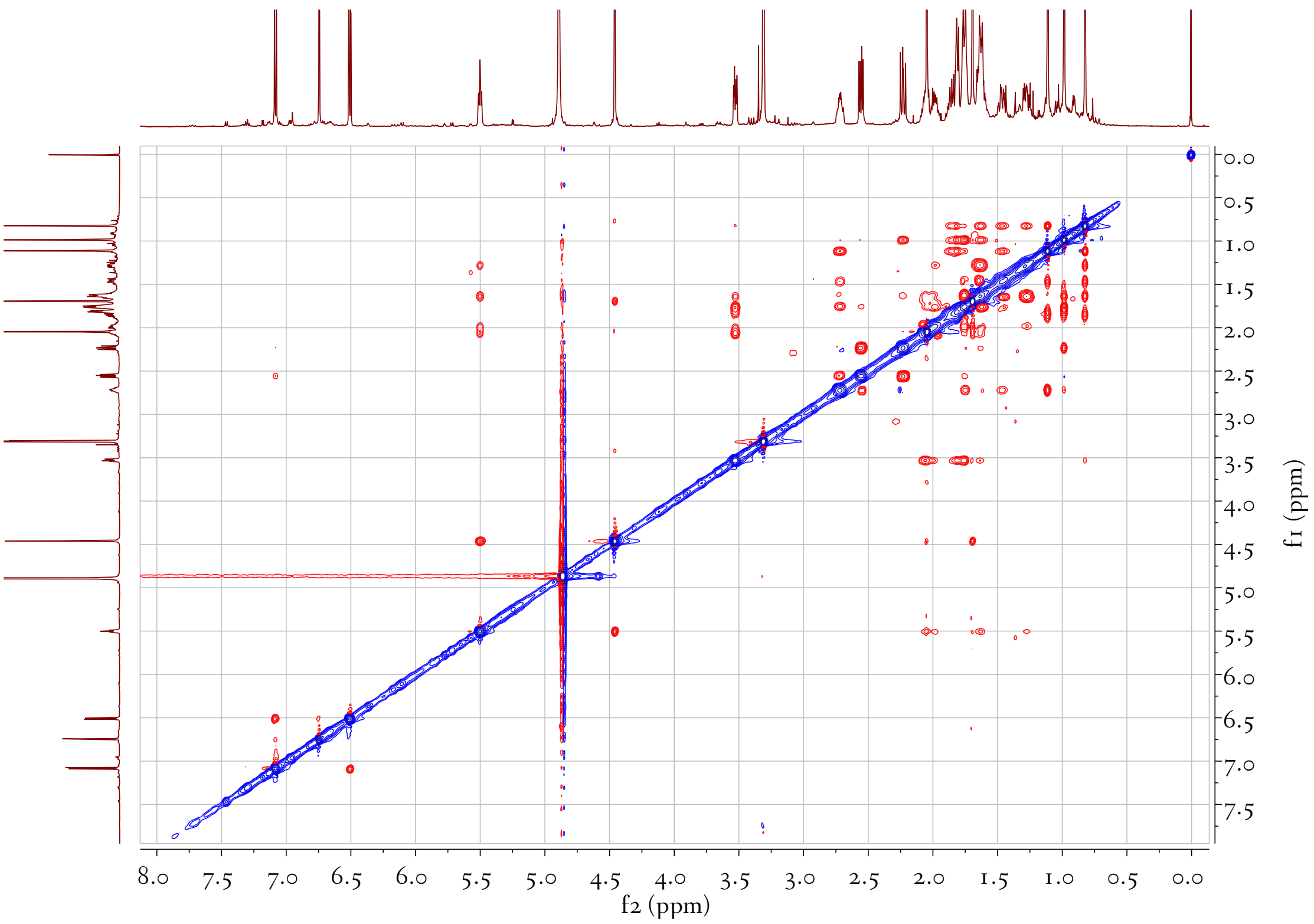

Figure S6. NOESY spectrum of **1**

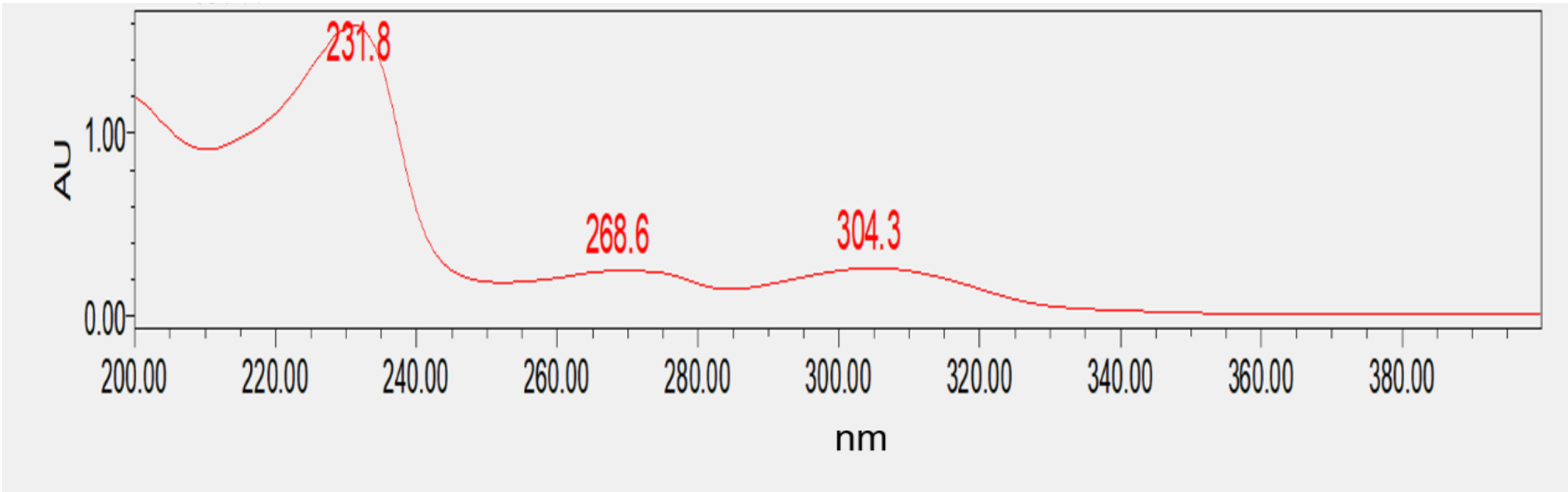

Figure S7. UV spectrum of **1** (100% MeOH/H_2O, extracted from HPLC-DAD data)

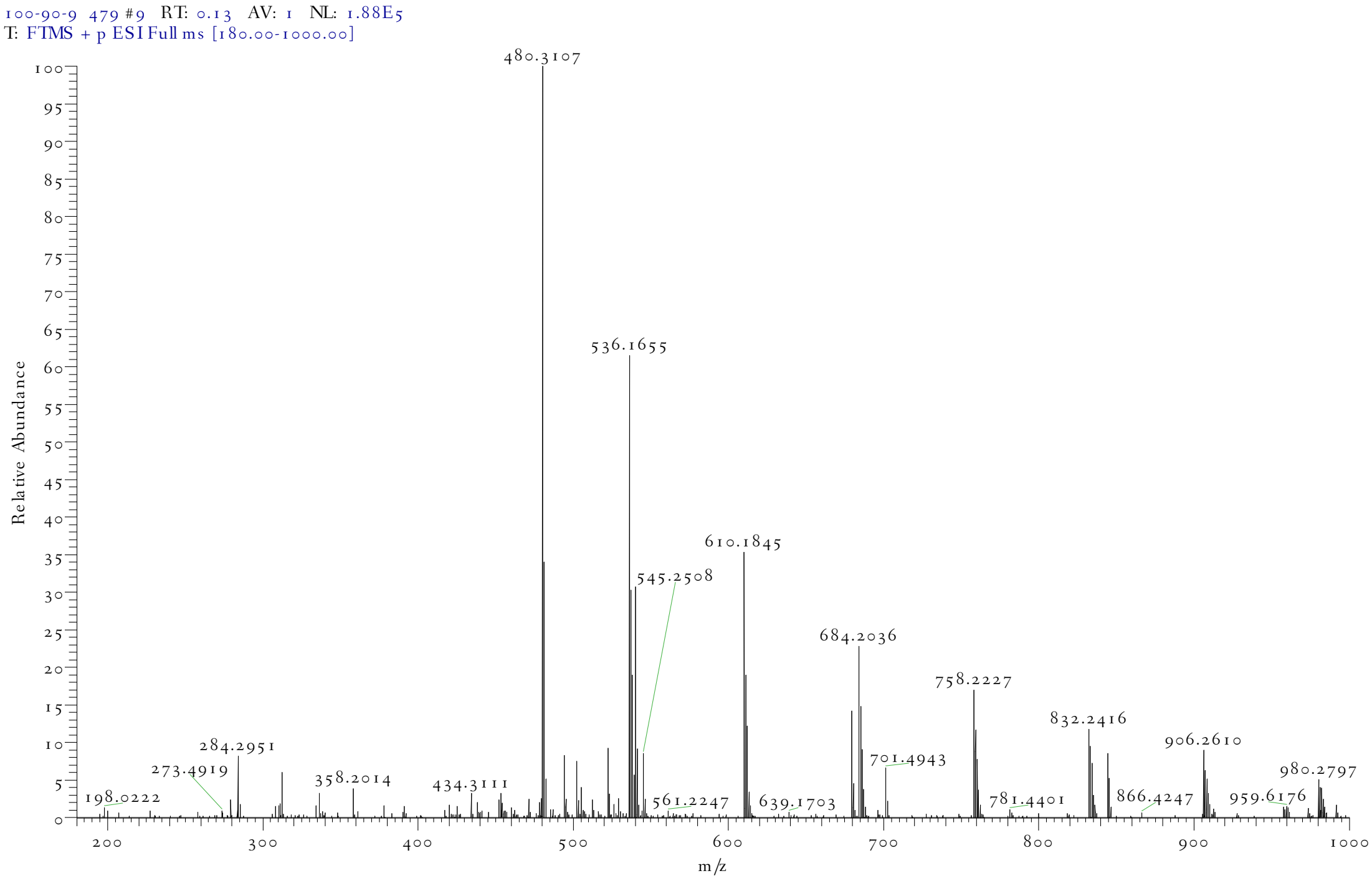

Figure S8. HRESIMS spectrum of **1**

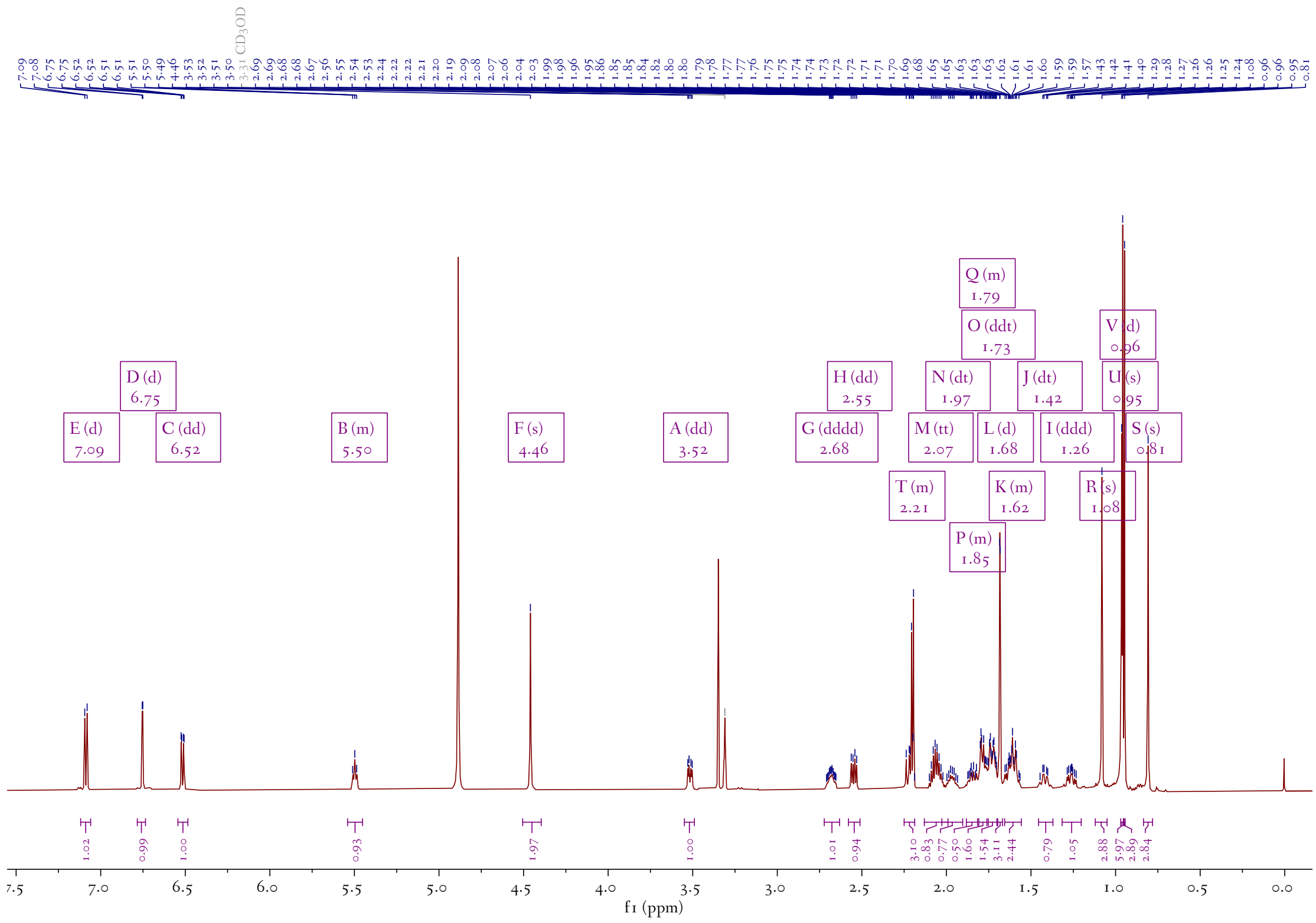

Figure S9. ^{1}H NMR spectrum of **2** (CD_3OD, 500 MHz)

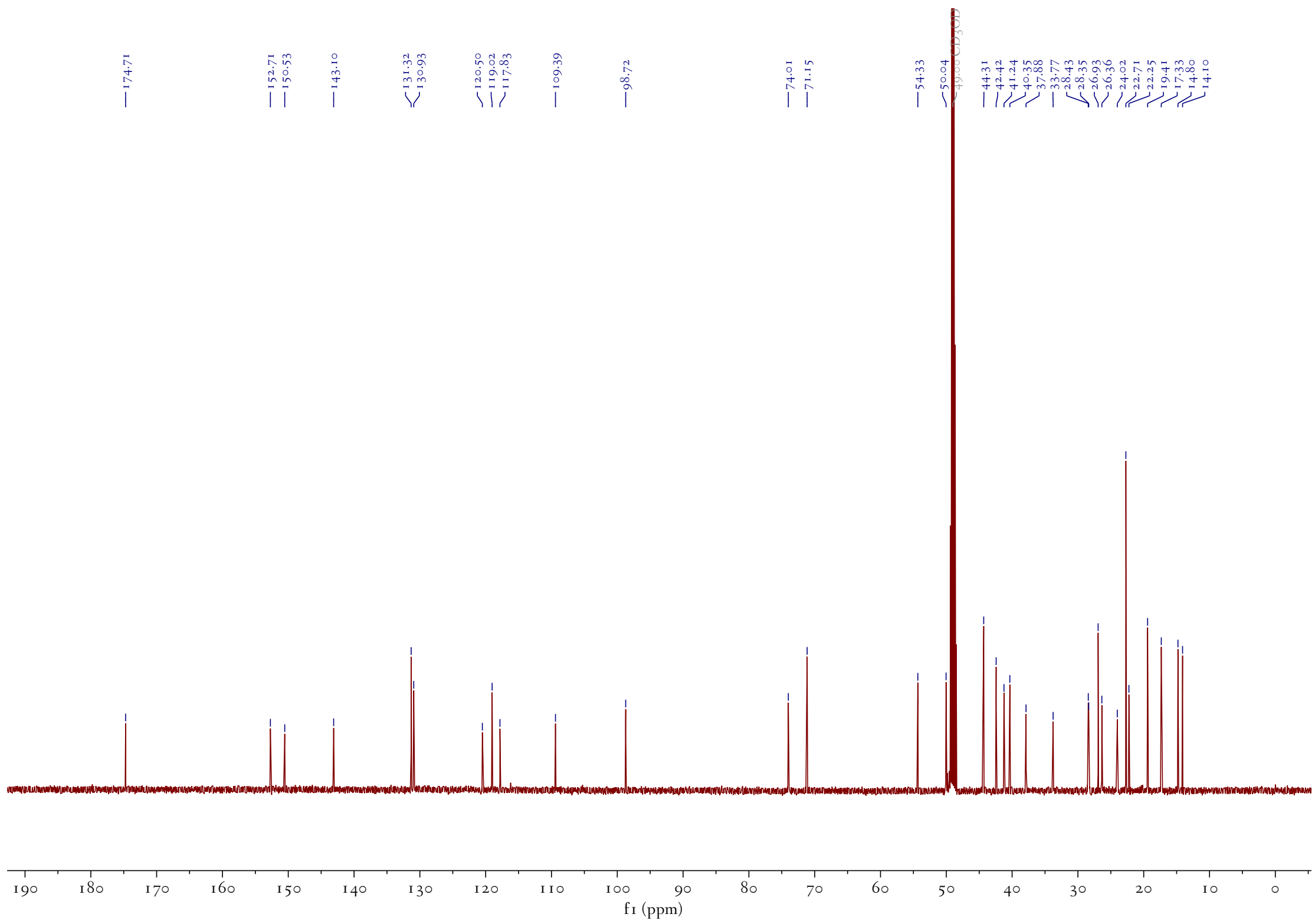

Figure S10. ^{13}C NMR spectrum of **2** (CD_3OD, 150 MHz)

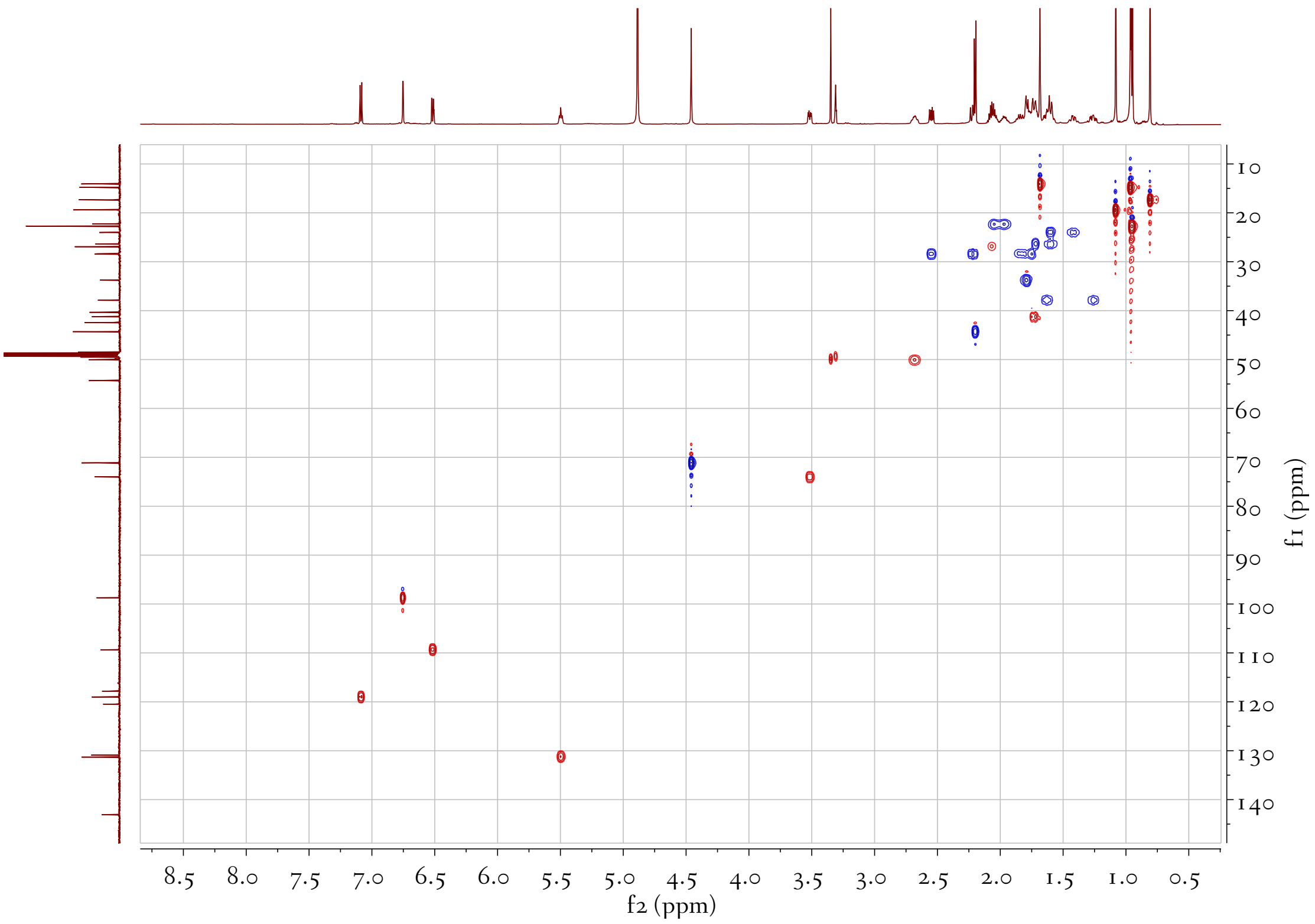

Figure S11. HSQC spectrum of **2**

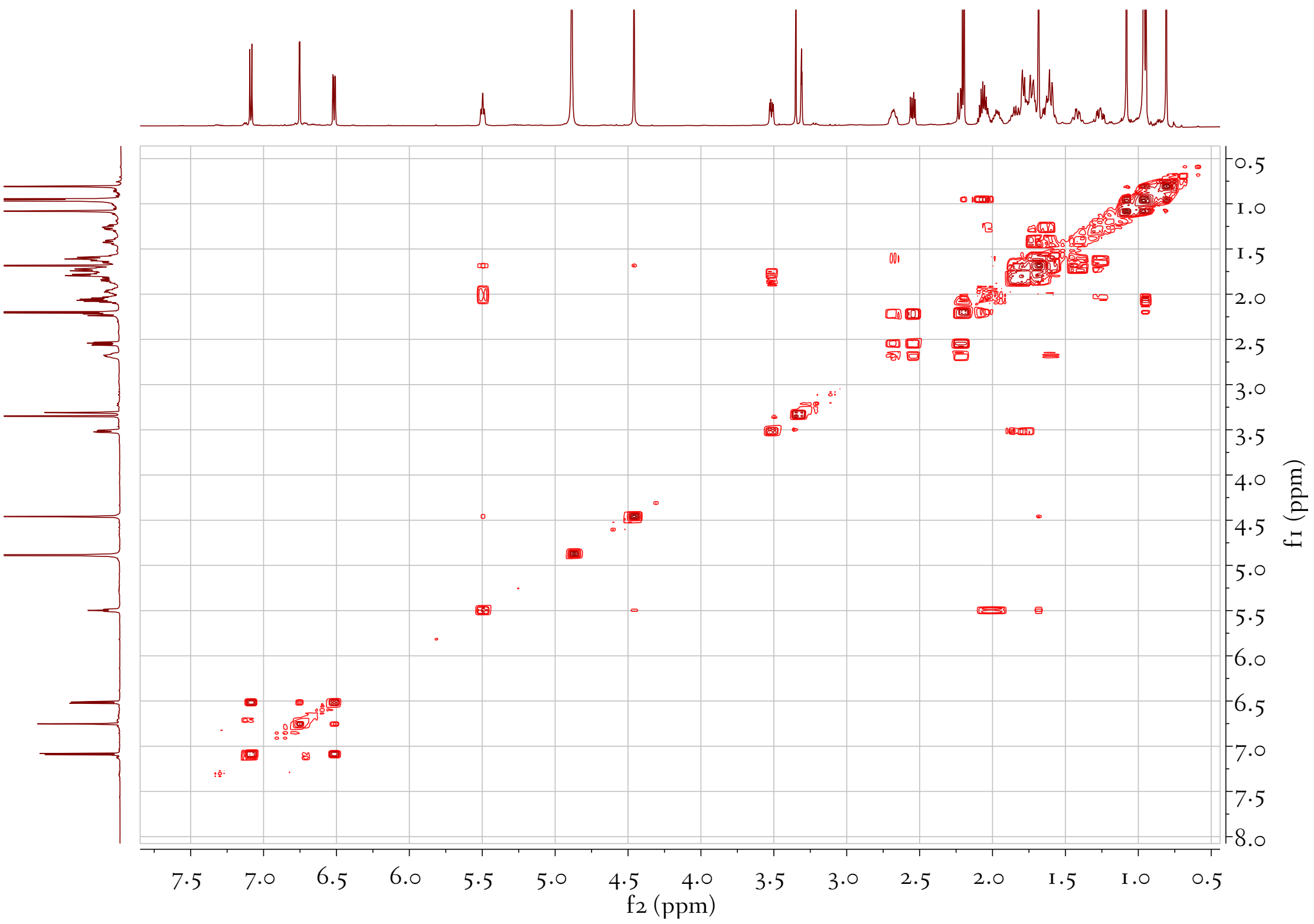

Figure S12. ^1H-^1H COSY spectrum of **2**

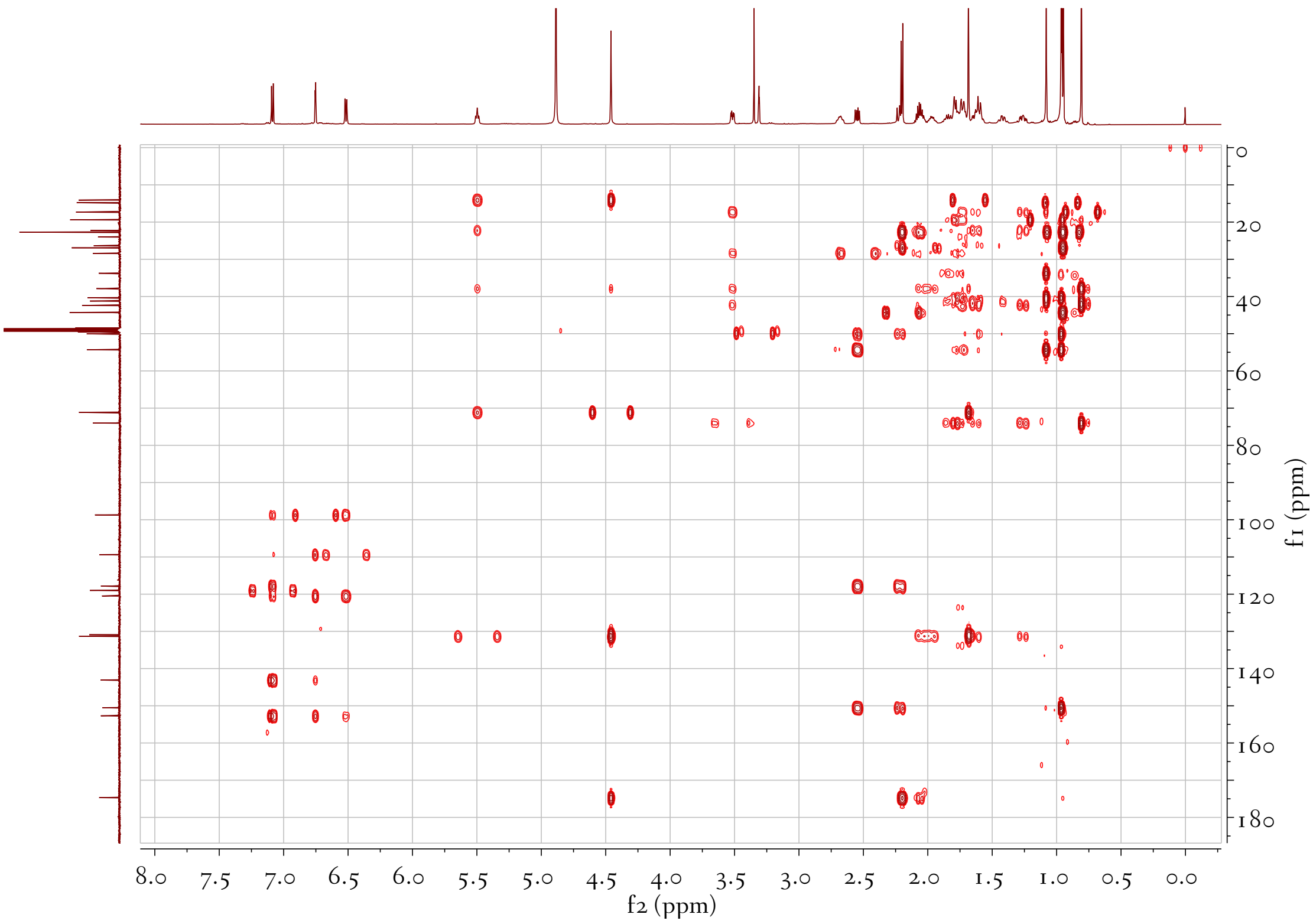

Figure S13. HMBC spectrum of **2**

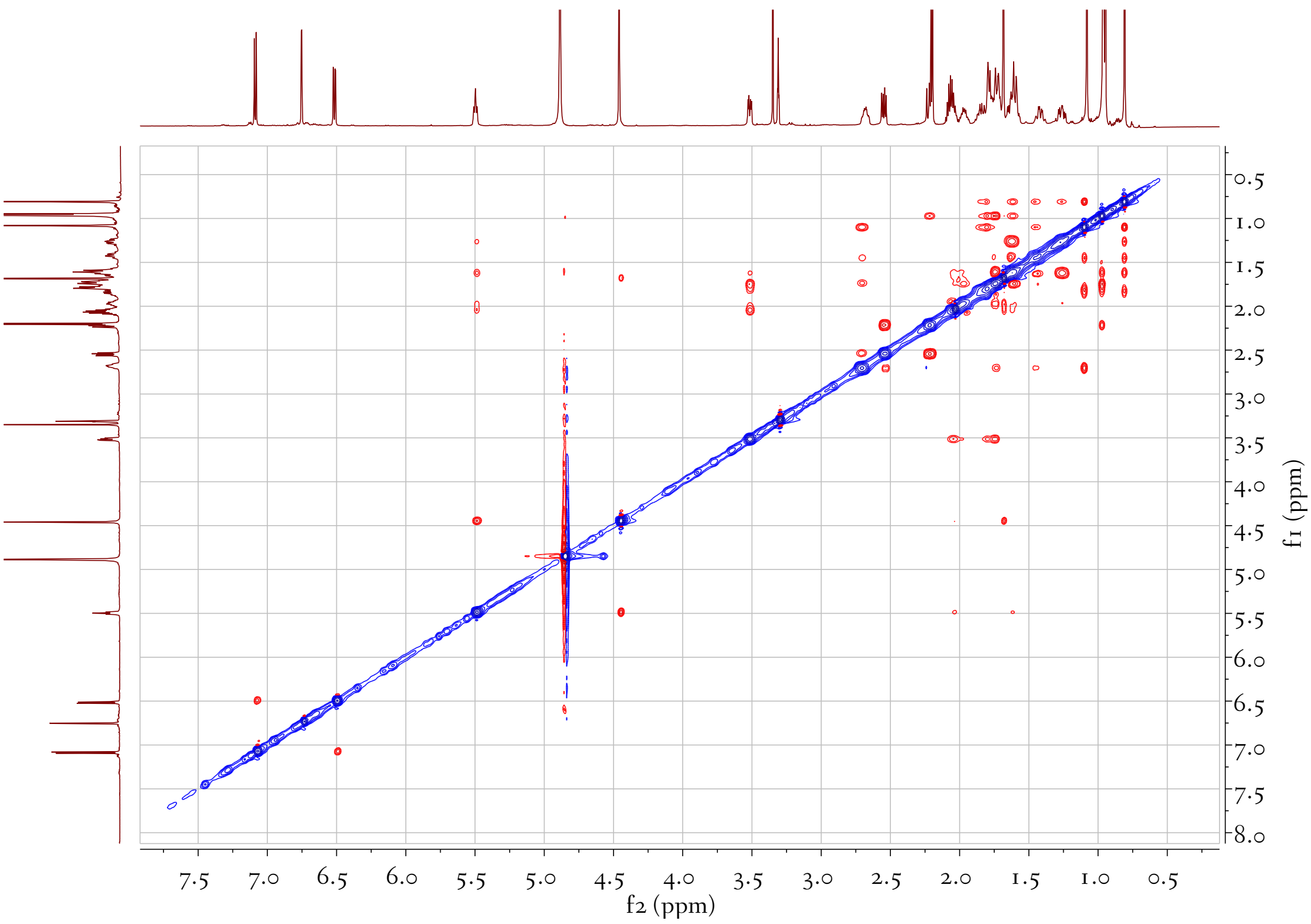

Figure S14. NOESY spectrum of **2**

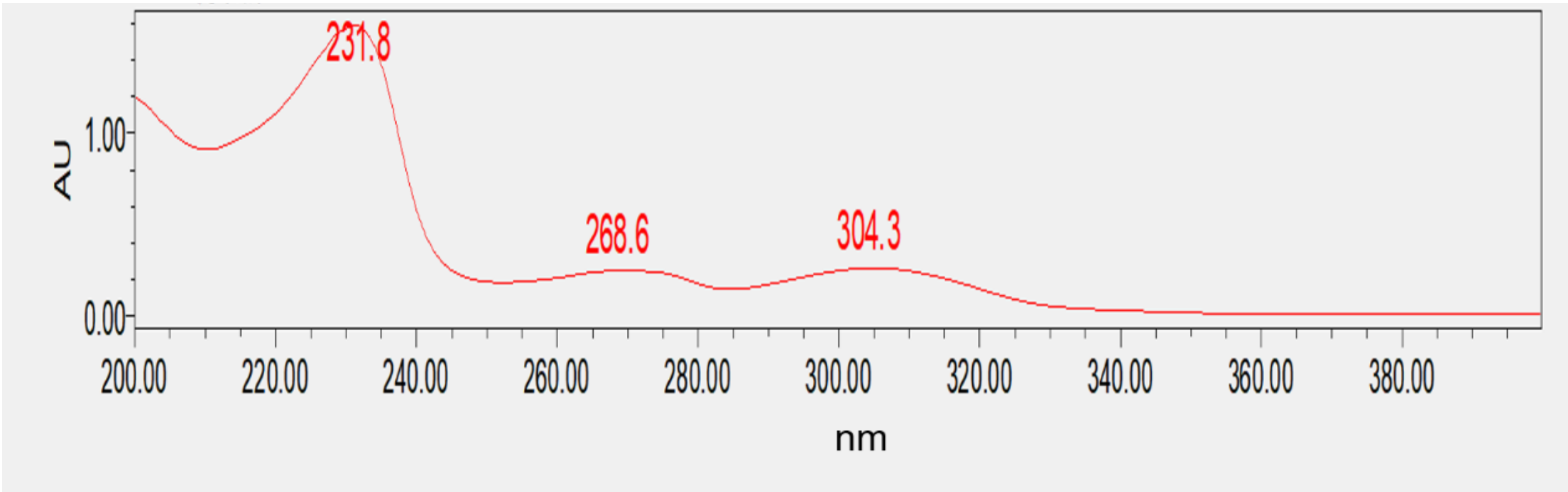

Figure S15. UV spectrum of **2** (100% MeOH/H_2O, extracted from HPLC-DAD data)

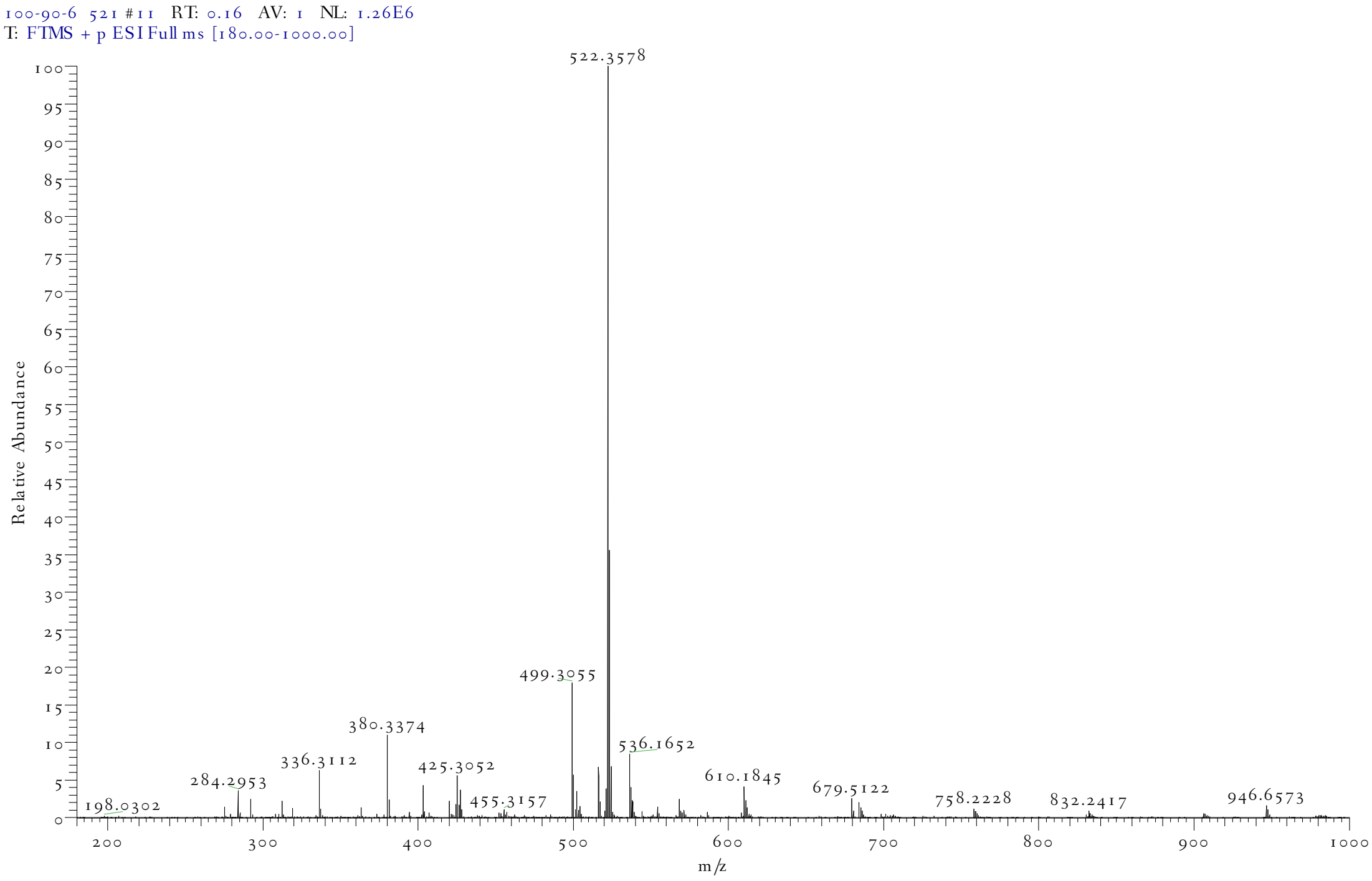

Figure S16. HRESIMS spectrum of **2**

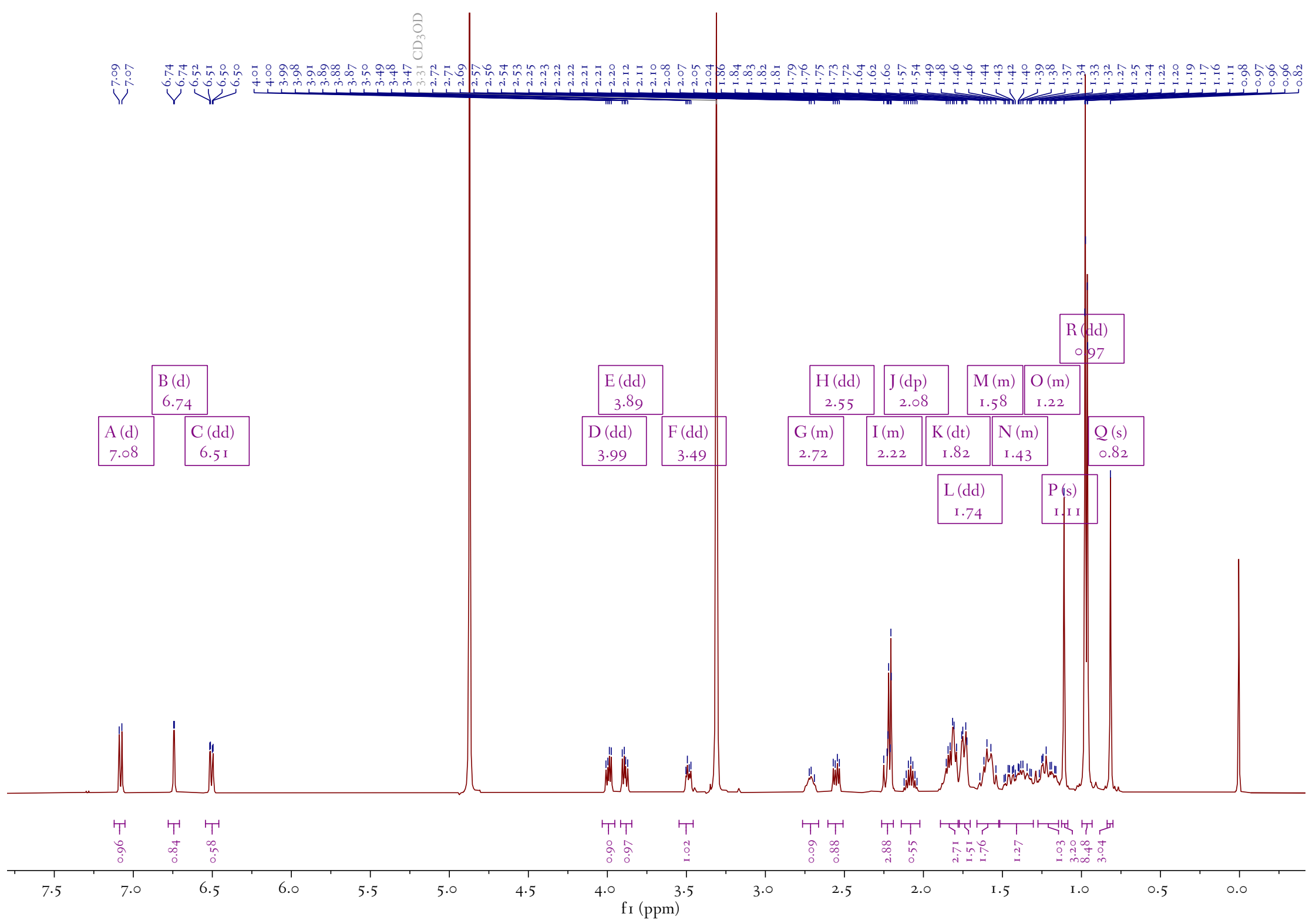

Figure S17. ^{1}H NMR spectrum of **3** (CD_3OD, 600 MHz)

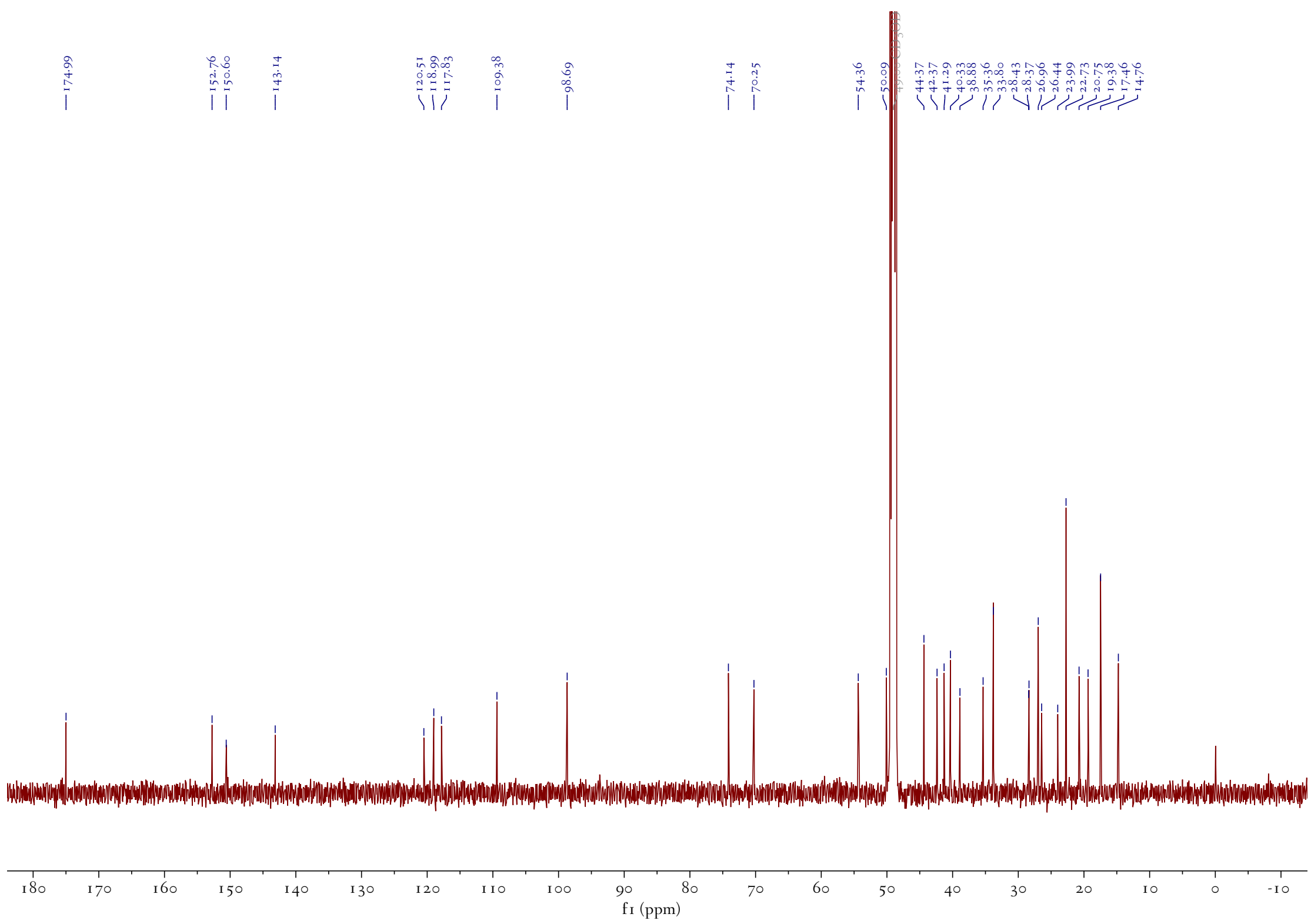

Figure S18. ^{13}C NMR spectrum of **3** (CD_3OD, 125 MHz)

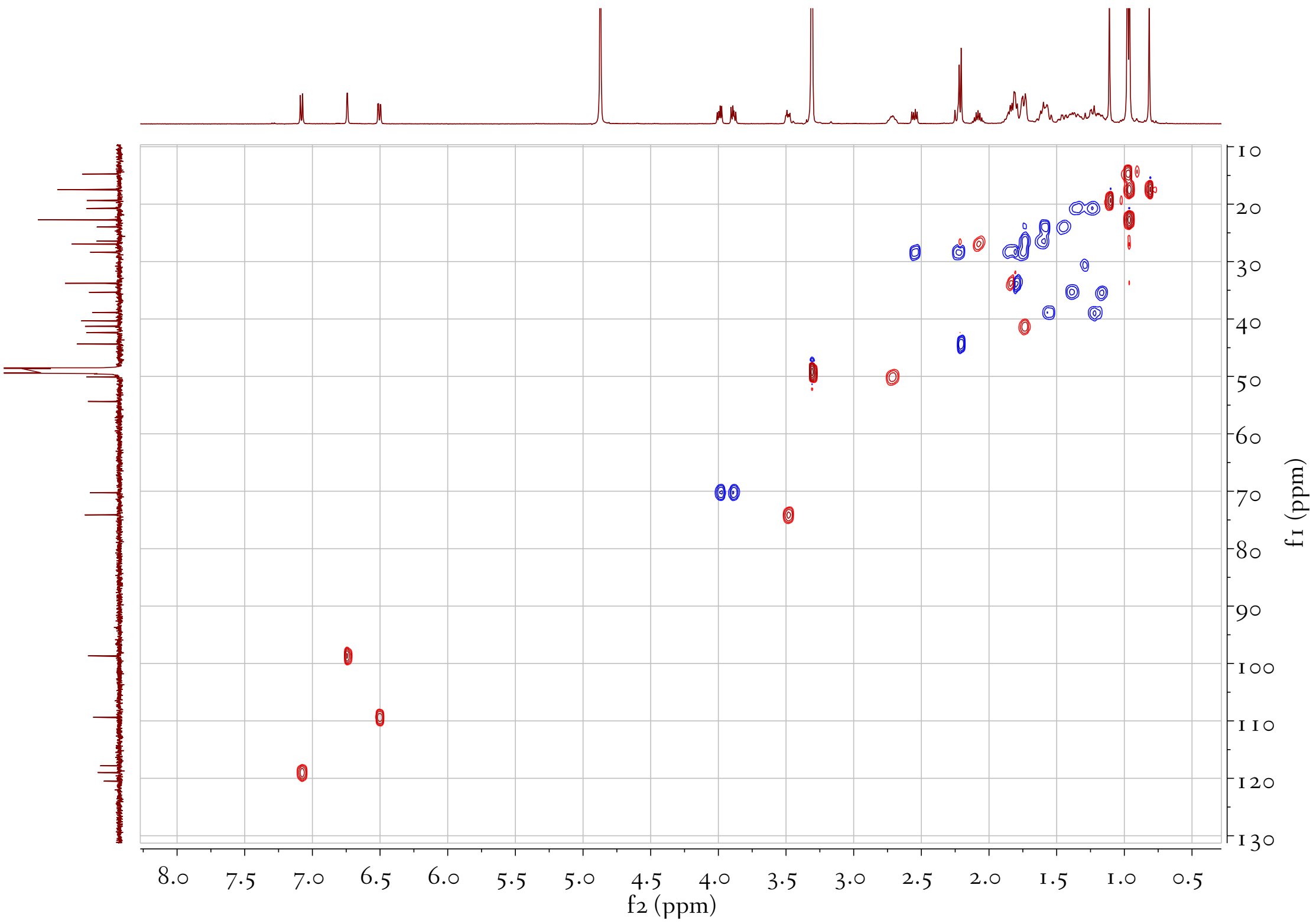

Figure S19. HSQC spectrum of **3**

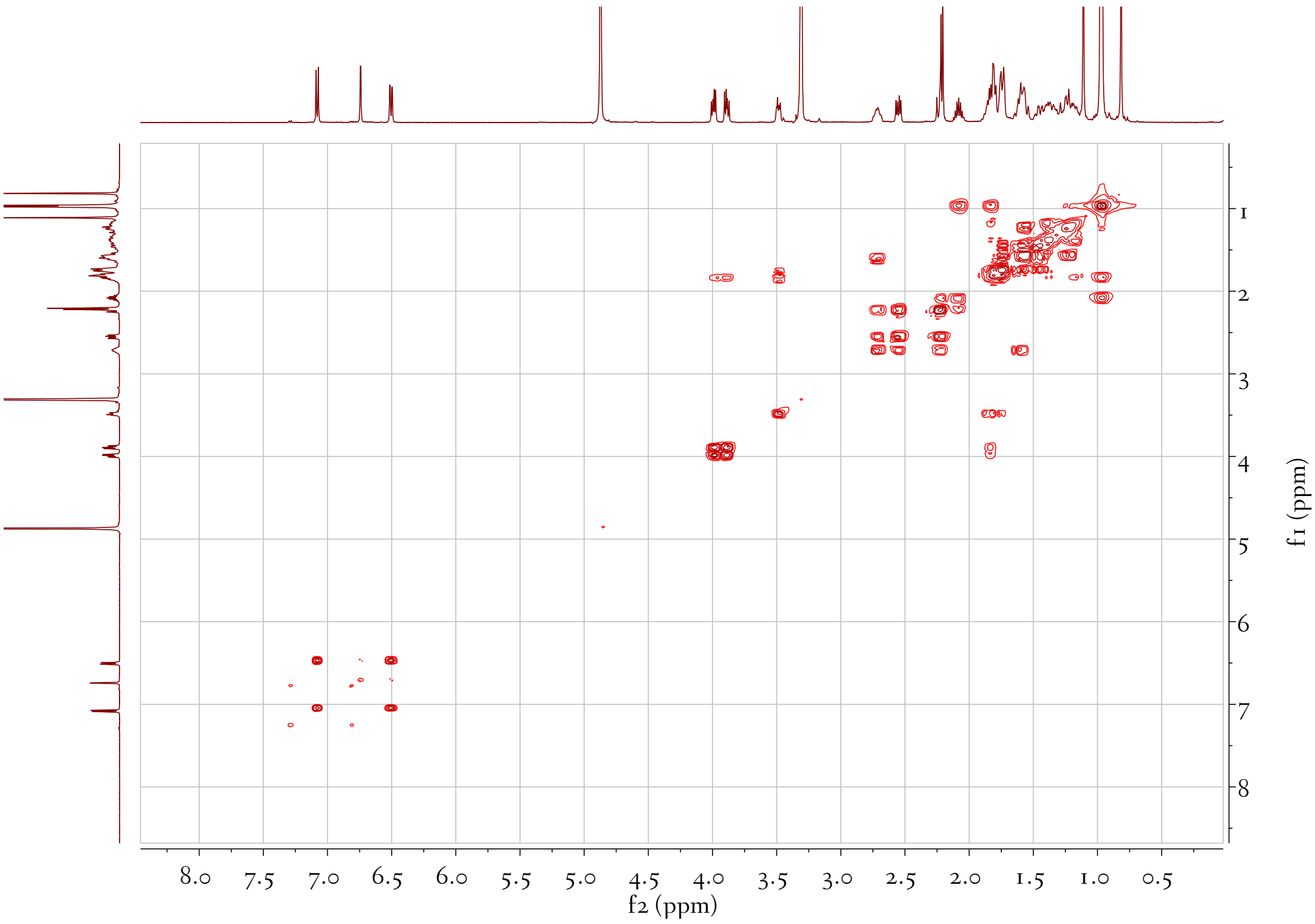

Figure S20. ^{1}H-^{1}H COSY spectrum of **3**

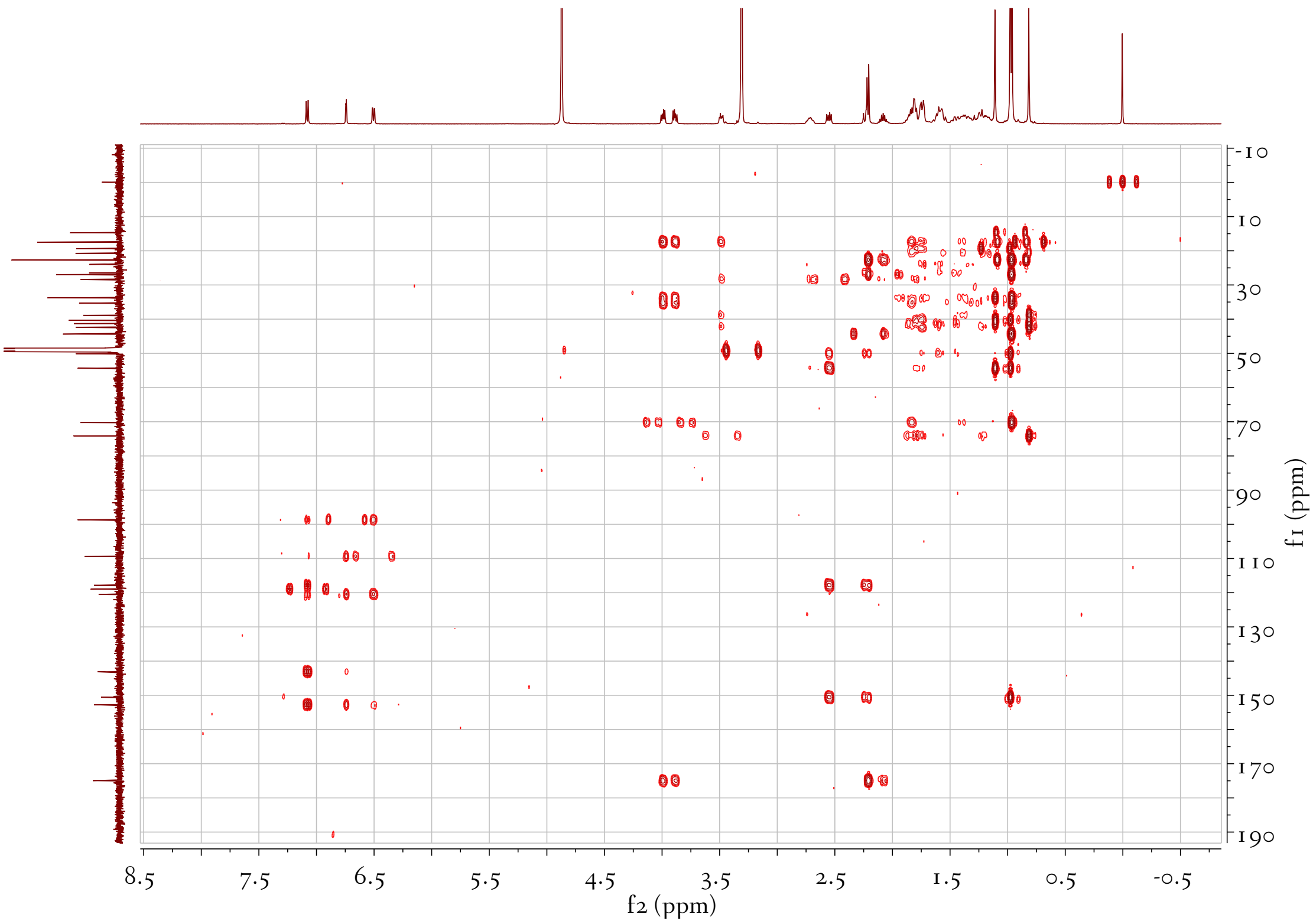

Figure S21. HMBC spectrum of **3**

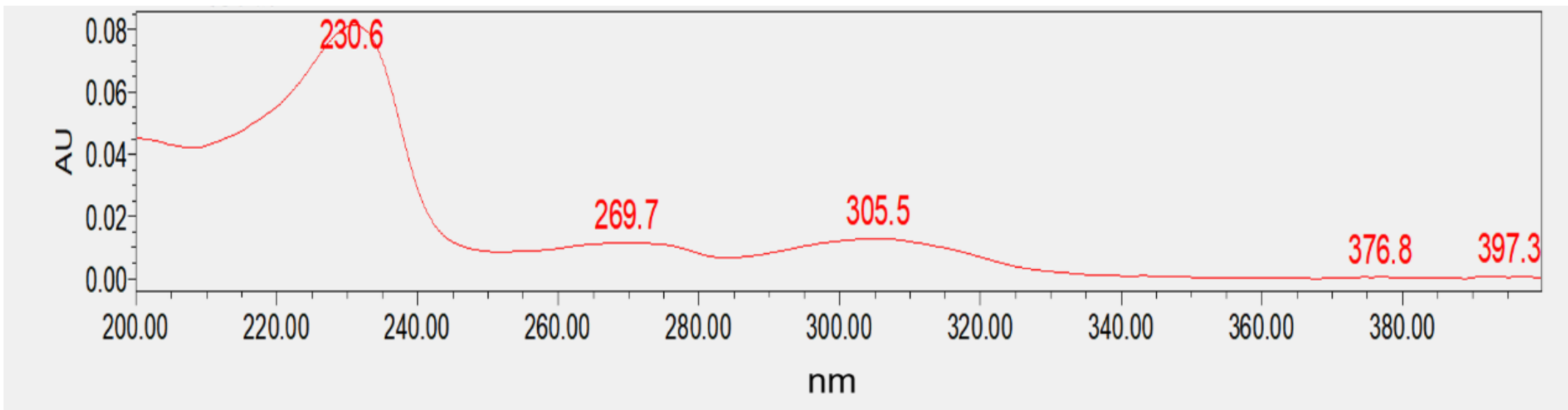

Figure S22. UV spectrum of **3** (100% MeOH/H_2O, extracted from HPLC-DAD data)

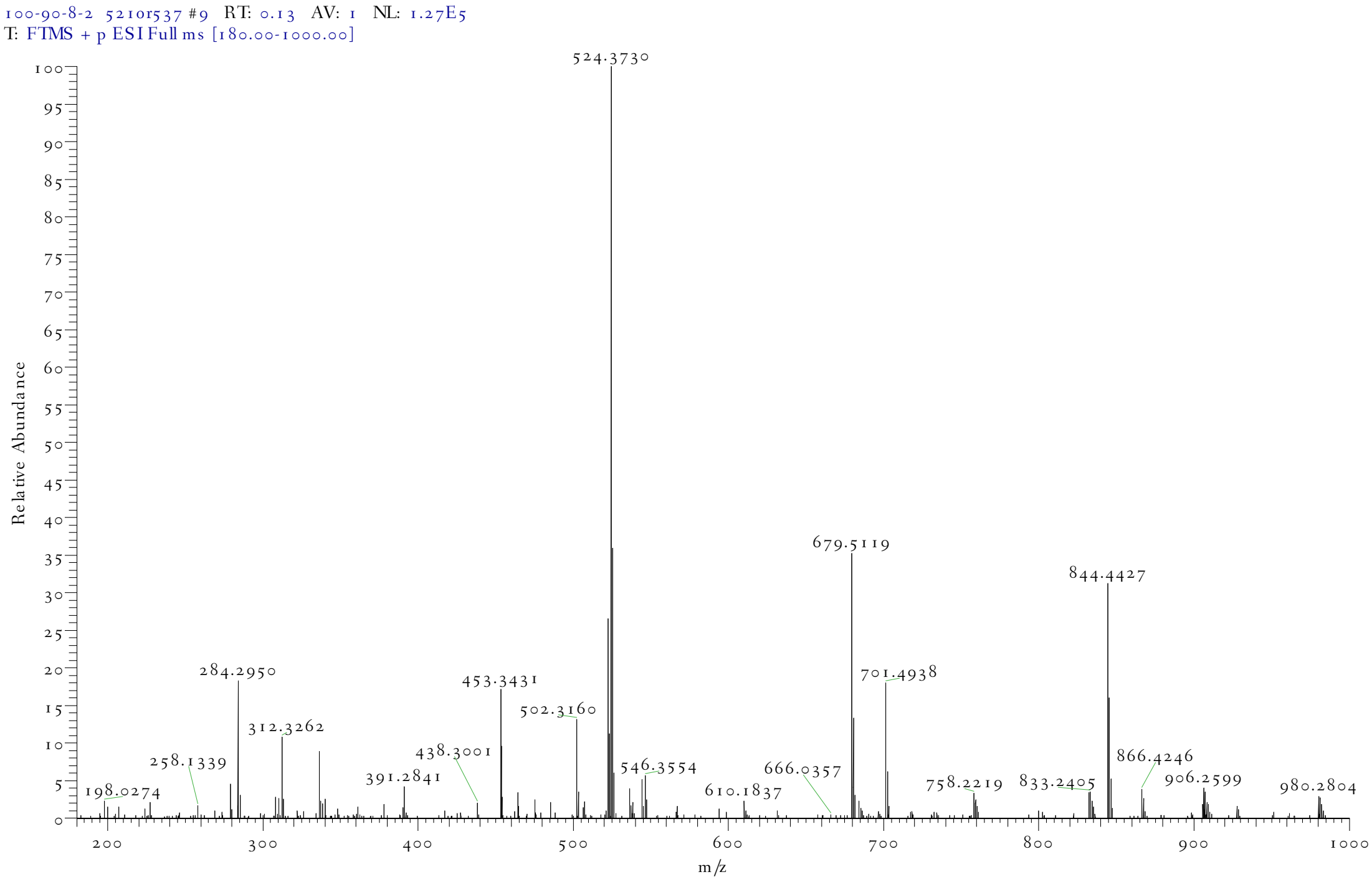

Figure S23. HRESIMS spectrum of **3**

Table S1. GIAO ^{13}C NMR calculation of two potential isomers of japonindolate C (**3**)

Position	Exp. δ_C	Cal. δ_C[a] (24*S**–**3**)	$\Delta\delta$ (cal. – exp.)	Cal. δ_C[a] (24*R**–**3**)	$\Delta\delta$ (cal. – exp.)
2	150.6	150.6	0.0	149.9	-0.7
3	117.8	118.1	0.3	119.2	1.4
4	120.5	119.0	-1.5	119.2	-1.3
5	119	118.0	-1.0	118.1	-0.9
6	109.4	106.1	-3.3	105.9	-3.5
7	152.8	151.8	-1.0	151.8	-1.0
8	98.7	96.5	-2.2	96.1	-2.6
9	143.1	139.9	-3.2	139.8	-3.3
10	28.4	28.8	0.4	29.2	0.8
11	50.1	49.2	-0.9	50.5	0.4
12	54.4	57.2	2.8	57.7	3.3
13	40.3	44.5	4.2	43.6	3.3
14	41.3	41.9	0.6	41.9	0.6
15	24.0	25.1	1.1	24.5	0.5
16	26.4	26.2	-0.2	26.8	0.4
17	33.8	33.8	0.0	34.1	0.3
18	28.4	29.5	1.1	28.9	0.5
19	74.1	71.0	-3.1	71.8	-2.3
20	42.4	46.0	3.6	46.9	4.5
21	38.9	35.9	-3.0	37.1	-1.8
22	20.8	18.9	-1.9	20.9	0.1
23	35.4	32.6	-2.8	33.4	-2.0
24	33.8	34.1	0.3	37.1	3.3
25	70.3	68.2	-2.1	66.1	-4.2
26	17.5	18.0	0.5	19.0	1.5
27	17.5	16.0	-1.5	16.5	-1.0
28	19.4	18.5	-0.9	18.5	-0.9
29	14.8	13.8	-1.0	14.1	-0.7
30	175.0	178.0	3.0	178.2	3.2
31	44.4	43.4	-1.0	43.0	-1.4
32	27.0	30.1	3.1	29.0	2.0
33	22.7	22.0	-0.7	21.9	-0.8
34	22.7	20.3	-2.4	20.6	-2.1
		RMSD	2.49	RMSD	2.58
		MAE	2.39	MAE	2.56

[a] ^{13}C NMR calculations were performed at the mpw1pw91/6-311+g(2d,p) (PCM=MeOH)//b3lyp/6-31+g(d,p) level, then cal. δ_C was scaling corrected from the calculated shielding tensor (σ) using the formula $\delta_C = (186.5993-\sigma)/1.0399$.

Table S2. ^{13}C NMR-based DP4+ analysis of the two candidate stereoisomers of japonindolate C (**3**): Isomer 1, 24*S*-3; Isomer 2, 24*R*-**3**

Functional		Solvent?		Basis Set		Type of Data	
mPW1PW91		PCM		6-311+G(d,p)		Scaled Shifts	
	Isomer 1	Isomer 2	Isomer 3	Isomer 4	Isomer 5	Isomer 6	
sDP4+ (H data)	-	-	-	-	-	-	
sDP4+ (C data)	78.14%	21.86%	-	-	-	-	
sDP4+ (all data)	78.14%	21.86%	-	-	-	-	
uDP4+ (H data)	-	-	-	-	-	-	
uDP4+ (C data)	-	-	-	-	-	-	
uDP4+ (all data)	-	-	-	-	-	-	
DP4+ (H data)	-	-	-	-	-	-	
DP4+ (C data)	-	-	-	-	-	-	
DP4+ (all data)	-	-	-	-	-	-	
